# A novel insight into mechanism of derangement of coagulation balance: interactions of quantum dots with coagulation-related proteins

**DOI:** 10.1186/s12989-022-00458-x

**Published:** 2022-03-08

**Authors:** Lingyan Zhang, Yingting Wu, Xingling Luo, Tianjiang Jia, Kexin Li, Lihong Zhou, Zhen Mao, Peili Huang

**Affiliations:** 1 School of Public Health, Capital Medical University, No. 10 Xitoutiao You An Men, Beijing, 100069 China; 2 School of Public Health, Baotou Medical College, 31# Jianshe Road, Donghe District, Baotou, 014040 China; 3 Core Facility Center, Capital Medical University, No. 10 Xitoutiao You An Men, Beijing, 100069 China

**Keywords:** Quantum dots, Interaction, Coagulation, Prothrombin, Plasminogen, Fibrinogen

## Abstract

**Background:**

Quantum dots (QDs) have gained increased attention for their extensive biomedical and electronic products applications. Due to the high priority of QDs in contacting the circulatory system, understanding the hemocompatibility of QDs is one of the most important aspects for their biosafety evaluation. Thus far, the effect of QDs on coagulation balance haven’t been fully understood, and limited studies also have yet elucidated the potential mechanism from the perspective of interaction of QDs with coagulation-related proteins.

**Results:**

QDs induced the derangement of coagulation balance by prolonging the activated partial thromboplastin time and prothrombin time as well as changing the expression levels of coagulation and fibrinolytic factors. The contact of QDs with PTM (prothrombin), PLG (plasminogen) and FIB (fibrinogen) which are primary coagulation-related proteins in the coagulation and fibrinolysis systems formed QDs-protein conjugates through hydrogen-bonding and hydrophobic interaction. The affinity of proteins with QDs followed the order of PTM > PLG > FIB, and was larger with CdTe/ZnS QDs than CdTe QDs. Binding with QDs not only induced static fluorescence quenching of PTM, PLG and FIB, but also altered their conformational structures. The binding of QDs to the active sites of PTM, PLG and FIB may promote the activation of proteins, thus interfering the hemostasis and fibrinolysis processes.

**Conclusions:**

The interactions of QDs with PTM, PLG and FIB may be key contributors for interference of coagulation balance, that is helpful to achieve a reliable and comprehensive evaluation on the potential biological influence of QDs from the molecular level.

**Supplementary Information:**

The online version contains supplementary material available at 10.1186/s12989-022-00458-x.

## Background

Quantum dots (QDs), also commonly known as semiconductor fluorescent nanoparticles, are the focus of extensive research in the field of nanoscience. Popular QDs contain a semiconductor core which typically consists of CdSe, CdTe, CdS and are often encapsulated in an outer shell (e.g., ZnS) [1–4]. Due to the unique size and shape dependent perfect optoelectronic properties, QDs are developed for a variety of application in biomedical imaging, drug delivery, solar cells, light emitting diodes as well as embedded in different display devices [5–9]. Nevertheless, QDs accumulated as well as released in various application will inevitably enter the bloodstream of living organisms at some point, thus causing the unintended harmful effects. Geys et al. found that CdSe/ZnS QDs may activate the coagulation cascade pathway through the contact with coagulation factor and induce pulmonary vascular thrombosis [10]. Samuel et al. demonstrated that 2.6 nm CdTe QDs were able to cause platelet aggregation in the absence of plasma, but in the presence of plasma, no platelet aggregation was found [11]. It was speculated that the interaction of plasma proteins with the QDs blocked the platelet aggregation induced by QDs. However, Maguire et al. presented contrasting results that 3.2 nm CdTe QDs may be able to interact with key coagulation factors that eliciting dramatic anticoagulant effects, whereas the 3.6 nm CdTe QDs stand above this critical size rendering them unable to bind crucial components [12]. The results mentioned above indicated that the nano-biointerface interaction of QDs with plasma proteins, especially coagulation-related proteins, may be a key contributor for interference of blood coagulation system homeostasis. QDs with various size and core/shell structure may also have inconsistent consequence in interaction with coagulation-related proteins.

Prothrombin (PTM, coagulation factor II), plasminogen (PLG, fibrinolytic factor) and fibrinogen (FIB, coagulation factor I) are key coagulation-related proteins in the process of hemostasis and fibrinolysis. PTM and PLG are two kinds of glycoprotein zymogens with molecular weight of 70 KDa and 92 KDa, respectively [13, 14]. PTM could be activated to form thrombin, which may not only convert fibrinogen into fibrin, but also activate plasminogen into plasmin, thus activating the fibrinolysis system. Severe deficiency of PTM is always characterized by severe bleeding, including prolonged post-injury bleeding, mucosal bleeding, and subcutaneous and muscle hematoma [14]. PLG is the inactive zymogen form of plasmin, which is capable of reducing the entire population of circulatory fibrinogen to degradation fragments within minutes, resulting in a generalized haemorrhagic state [15]. Murine knockout studies reveal that plasminogen-deficient mice develop spontaneous fibrin deposition and severe thrombosis [16]. FIB is a homodimeric glycoprotein with molecular weight of 340 KDa and an important substrate for thrombosis in the process of coagulation and hemostasis [17]. The main function of FIB is to form fibrin clots and thereby stop excessive bleeding caused by tissue and vascular injury [18]. The interactions of these specific coagulation-related proteins with nanoparticles (NPs) may not only induce the changes of conformational structures and biological functions of proteins, but also crucially determine the biological fate of NPs [19–22]. However, limited comprehension have yet elucidated the interference mechanism of the interaction of QDs with key coagulation-related proteins on coagulation homeostasis.

In this study, we investigated the effects of CdTe QDs and CdTe/ZnS QDs on coagulation balance in rats and the interacting mechanism of CdTe QDs and CdTe/ZnS QDs with FIB, PLG and PTM. The effects of QDs on coagulation balance were evaluated by measuring prothrombin time (PT), activated partial thromboplastin time (APTT), thrombin time (TT) and the expression level of coagulation factors, anticoagulation factors and fibrinolytic factors. Meanwhile, capillary electrophoresis connected to inductively coupled plasma mass spectrometry (CE-ICP-MS) monitored the formation of QDs-protein conjugates. Isothermal titration calorimetry (ITC) and bio-layer interferometry (BLI) were used for the basic kinetic and thermodynamic study of interaction of QDs with FIB, PLG and PTM. Fluorescence spectra and circular dichroism (CD) spectra assayed the fluorescence quenching of both proteins and QDs after interaction, and the conformational changes of proteins upon binding with QDs. Molecular docking analyses displayed the specific binding sites and the binding forces of the modified groups L-glutathione (L-GSH) and L-cysteine (L-Cys) of QDs with the active pockets of FIB, PLG and PTM. We expected to obtain novel insights for QDs interference with coagulation balance, that may not only provide new hints to achieve a reliable and comprehensive evaluation on the biosafety of QDs, but also contribute data support for guiding the design of highly secured QDs.

## Results and discussion

### Characterization of CdTe QDs and CdTe/ZnS QDs

The absorption and emission maximum peaks for the QDs are centered at 526 nm, 566 nm (CdTe QDs) and 550 nm, 588 nm (CdTe/ZnS QDs) (Additional file 1: Fig. S1). A well-resolved absorption maximum of the first electronic transition and narrow fluorescence spectrum were observed, pointing to a monodispersed and homogeneous size distribution of the prepared CdTe QDs and CdTe/ZnS QDs. The particle-size distribution and morphology of CdTe QDs and CdTe/ZnS were measured by transmission electron microscopy (TEM) (Additional file 1: Fig. S2). The estimated diameters obtained for CdTe QDs and CdTe/ZnS QDs were of 2.91 nm and 3.24 nm according to calculate method in literature, respectively [23]. This gave the thickness of the ZnS shell as ∼ 0.3 nm. Regarding the zeta potential, the CdTe/ZnS QDs (− 56.6 ± 4.2 mV) are more stable than the CdTe QDs (− 41.2 ± 3.6 mV).

### QDs induced derangement of coagulation balance

**Fig. 1:**
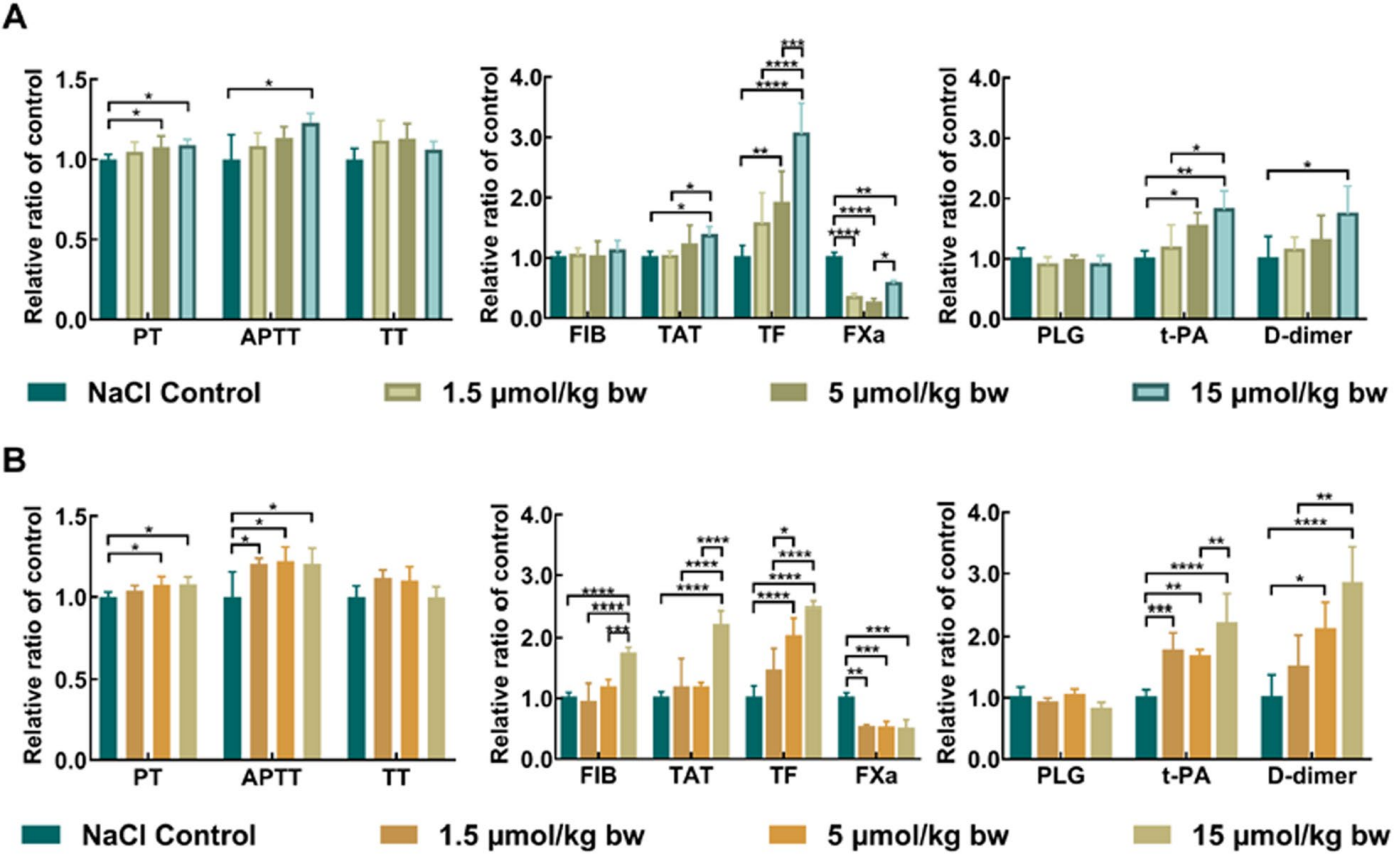
Effects of CdTe QDs (**A**) and CdTe/ZnS QDs (**B**) on coagulation function, coagulation factors and fibrinolytic factors. The data for each group of coagulation function, coagulation factors and fibrinolytic factors were normalized by dividing by the mean of the corresponding control group. Each value represents the mean ± standard deviation (SD) (*n* = 6). * *P* < 0.05, ** *P* < 0.01, *** *P* < 0.001, **** *P* < 0.0001, comparing to the values of control group and dose group by two-way ANOVA with Bonferroni’s multiple comparison test

At days 1, 3, 7 and 14 after exposure of CdTe QDs and CdTe/ZnS QDs, PT, APTT and TT were measured to characterize the effects of CdTe QDs and CdTe/ZnS QDs on coagulation function in rats. As shown in Fig. 1, PT and APTT were prolonged in a dose-dependent manner at day 1, indicating that CdTe QDs and CdTe/ZnS QDs activated both intrinsic and extrinsic coagulation cascade pathways. The effects on APTT in 1.5 μmol/kg bw group and 5 μmol/kg bw group of CdTe/ZnS QDs were greater than CdTe QDs. Nevertheless, the effects on PT, APTT and TT disappeared in a short period of time (shorter than 3 days) for both CdTe QDs and CdTe/ZnS QDs (Additional file 1: Fig. S3). These results illustrated that exposure of CdTe QDs and CdTe/ZnS QDs caused coagulation function disorders in rats in a dose-dependent manner and time-dependent manner.

For further elucidating how coagulation function disorders were initiated by CdTe QDs and CdTe/ZnS QDs, factors screening was also carried out to identify the changes in the expression level of coagulation factors (FIB, TAT, TF, FXa), anticoagulation factors (TFPI, AT-III) and fibrinolytic factors (PLG, t-PA, D-dimer) in rats. As shown in Fig. 1, at day 1 after exposure, the interference of QDs with coagulation factors and fibrinolytic factors not only activated the coagulation cascade (e.g. FIB, TAT and TF obviously increased in a dose-dependent manner), but also exceeded the regulation ability of the natural coagulation and anticoagulation system [24]. Extensive production of thrombin and large consumption of coagulation factors (e.g. FXa significantly decreased) subsequently stimulated secondary fibrinolytic hyperactivity (e.g. D-dimer and t-PA remarkably increased in a dose-dependent manner). At day 3 and 7, the coagulation functions (PT and APTT) and some coagulation factors (FIB, TAT, TF) have recovered, but sustained consumption of FXa and PLG and generation of t-PA indicated that the fibrinolysis system was still activated to promote the microthrombus dissolution. Notably, the effects of CdTe/ZnS QDs on coagulation factors and fibrinolytic factors were stronger and lasted longer than CdTe QDs at same dosage (Additional file 1: Fig. S4, S5). For anticoagulation factors, no significant changes were observed for TFPI and AT-III in each dosage group of CdTe QDs and CdTe/ZnS QDs at each point in time of observation (Additional file 1: Fig. S6). These results illustrated that exposure of CdTe QDs and CdTe/ZnS QDs induced derangement of coagulation factors and fibrinolytic factors, that eventually caused coagulation function disorders. The core/shell structure and size of QDs may play a role in the coagulation balance. FIB, PLG and PTM, as initial and primary factors in the coagulation and fibrinolysis systems, their interaction with QDs may be key contributors for interference with coagulation balance.

### CE-ICP-MS to monitor the formation of QDs-protein conjugates

**Fig. 2:**
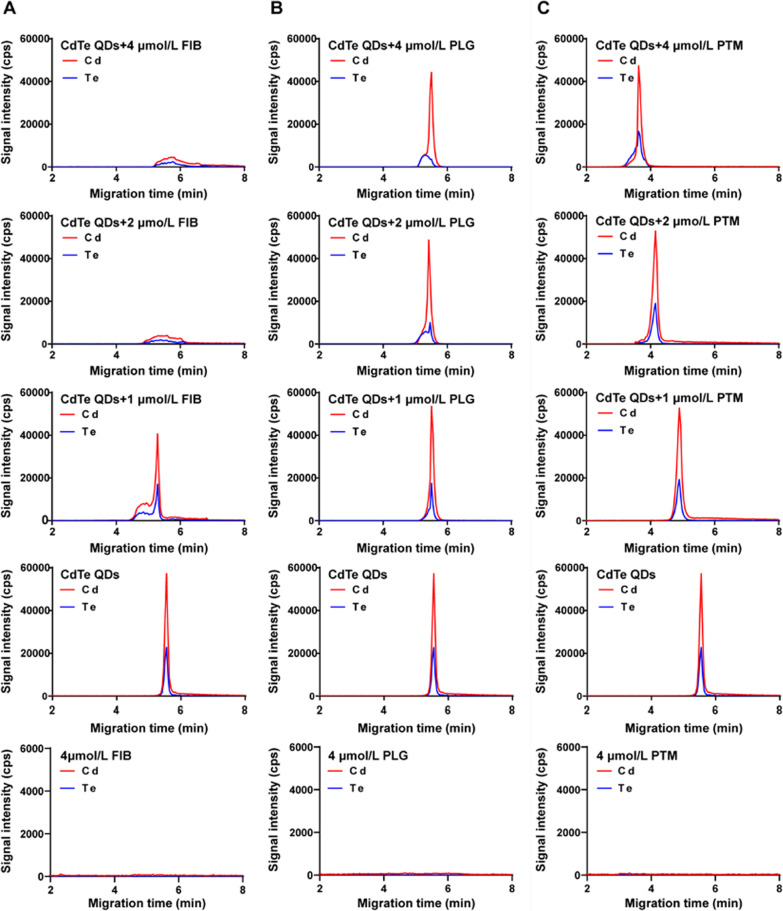
Electropherograms for interaction of CdTe QDs with FIB (**A**), PLG (**B**) and PTM (**C**). The concentrations of proteins were 0 μmol/L, 1 μmol/L, 2 μmol/L and 4 μmol/L, respectively. The concentration of CdTe QDs was 50 μmol/L

**Fig. 3:**
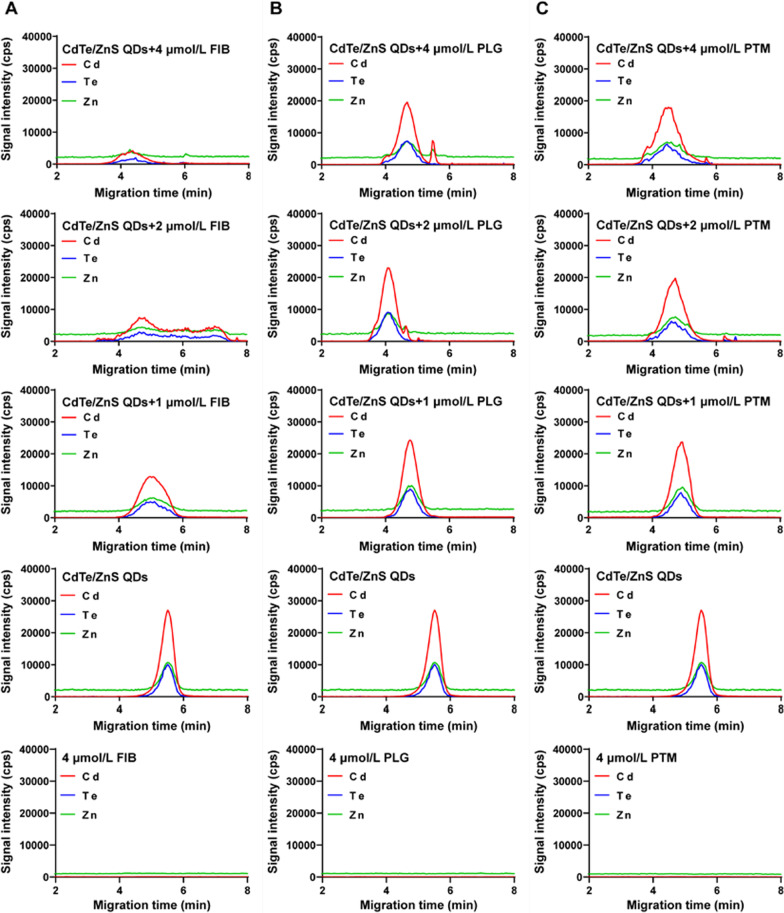
Electropherograms for interaction of CdTe/ZnS QDs with FIB (**A**), PLG (**B**) and PTM (**C**). The concentrations of proteins were 0 μmol/L, 1 μmol/L, 2 μmol/L and 4 μmol/L, respectively. The concentration of CdTe/ZnS QDs was 50 μmol/L

CE-ICP-MS is well known not only as an efficient separation technique but also as a viable tool to characterize the formation of metal-containing nanoscale materials upon interaction with biomolecules [25–28]. As shown in Figs. 2 and 3, 50 μmol/L CdTe QDs and CdTe/ZnS QDs were incubated respectively with different concentrations of FIB, PLG and PTM for 1 h, and the mixture was injected into the capillary. While the concentration of FIB reached 1 μmol/L, CdTe QDs emerge out of the capillary (moving toward the anode) at a notably shorter time and a new peak appeared. Along with higher FIB concentrations (≥ 2 μmol/L), the change in mobility was no longer observable, and a broader peak formed. For interaction with PLG, the peak slightly shifted to the left and the peak area of new peak increased with PLG concentration from 1 to 4 μmol/L. For interaction with PTM, the migration time of CdTe QDs shortened quickly with the concentration of PTM increased. A new peak did not appear until the concentration of PTM reached 4 μmol/L. These results suggested that the interaction of proteins with CdTe QDs formed QDs-protein conjugates, that bring in changes in charge and size of CdTe QDs, thus changing their mobility. The reduction in migration time of CdTe QDs after incubation with proteins was probably due to the increase in CdTe QDs size after being bound with proteins. The numbers of CdTe QDs-protein conjugates increased with protein concentration until conjugates formed enough and the dissociation rate was slower than the CE speed, while the stable QDs-protein conjugates were resolved from the free that caused the appearance of a new peak. When the concentration of FIB was larger than 4 μmol/L, the further aggregation of CdTe QDs-protein conjugates and the adsorption of excessive proteins on capillary wall may contribute to a broader peak and weak signal intensity in electropherograms for CdTe QDs [26].

Compared to CdTe QDs, the migration time of the whole peak shortened and the peak broadened for CdTe/ZnS QDs when the concentration of three proteins increased, but no new peak of QDs-protein conjugates appeared. These phenomena may be due to that thermodynamically stable CdTe/ZnS QDs-protein conjugates may form and attain an equilibrium state more rapidly even at a low concentration of proteins on account of higher affinity of CdTe/ZnS QDs with proteins than CdTe QDs. As the concentration of proteins increased continuously, the peak of CdTe/ZnS QDs-protein conjugates broadened gradually, indicating the aggregation of conjugates may occur. Besides, it is worth noting that a new Cd peak and a new Zn peak appeared at 2 μmol/L and 4 μmol/L PLG, that may be attribute to the degradation of CdTe/ZnS core/shell structure after binding with PLG. Several literatures have demonstrated that the surface exchange reaction between proteins and ligands on the surface of QDs may lead to the degradation of QDs, resulting in the release of Cd^2+^ and Zn^2+^ [29, 30]. Matczuk etc. have found that the ZnS shell of CdSeS/ZnS QDs have been removed after binding with an unknown protein with high molecular-mass in the serum [28]. Besides, Dhanya etc. also have found that NPs subjected to flow have a greater concentration of proteins absorbed on the surface, especially for PLG [31]. They also showed that in response to flow and binding to NPs surface may give rise to the structural changes of PLG, that was also consistent with our finding in CD spectra experiments.

### Kinetic and thermodynamic study of QDs-protein interactions

**Fig. 4:**
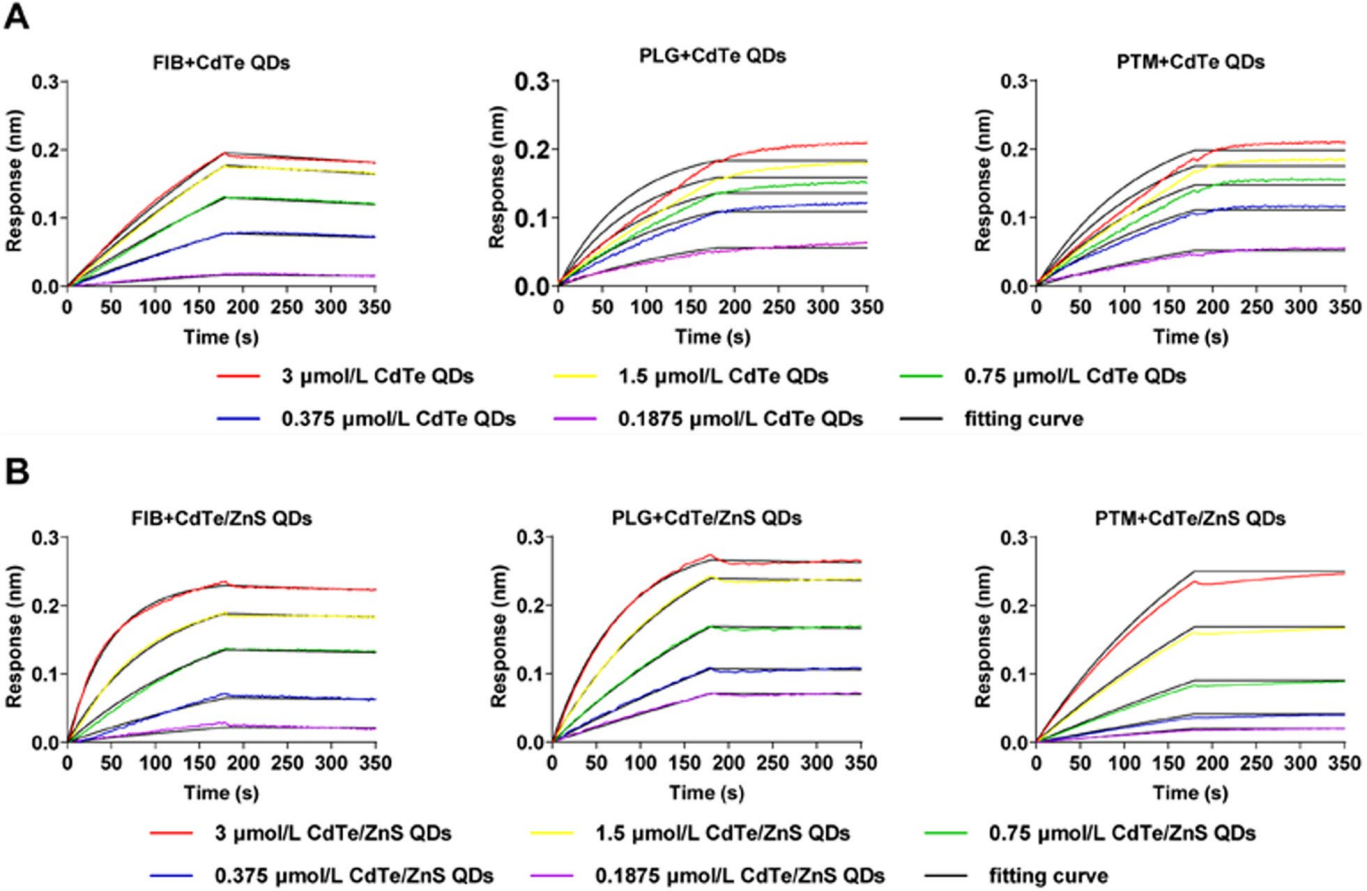
Association and dissociation curves for interaction of FIB, PLG and PTM with CdTe QDs (**A**) and CdTe/ZnS QDs (**B**)

Typical BLI assays were established for individual binding kinetics of the interaction of QDs with FIB, PTM and PLG, respectively (Fig. 4). During the association period from 0 to 180 s, with the increase of QDs concentrations and binding time, the number of QDs bound to the proteins immobilized on the SA biosensor tips increased, inducing the increase of thickness of the bio-layer. This concentration-dependent and time-dependent binding behavior of QDs and proteins caused the positive shifts of interference wavelength. During the dissociation period from 180 to 360 s, partial QDs dissociated from the protein, and the interference wavelength showed small negative shifts for dissociation of FIB with QDs whereas slightly positive shifts for dissociation of PLG and PTM with QDs. The inconsistency of dissociation curves and corresponding fitted curves illustrated that the interaction of QDs with PLG and PTM may result in the conformational changes of proteins and aggregation of QDs that caused the increase in thickness of the bio-layer and subsequent positive shift of interference wavelength. The *K*_a_ and *K*_dis_ for interaction of QDs and protein were obtained by fitting the response of association and dissociation on concentration of proteins and summarized in Table 1. The *K*_a_ for interaction of both CdTe QDs and CdTe/ZnS QDs with proteins followed the order of PLG > PTM > FIB. For the same protein, the *K*_a_ of CdTe/ZnS QDs was larger than CdTe QDs. Nevertheless, the accurate *K*_dis_ for interaction of both CdTe QDs and CdTe/ZnS QDs with PLG and PTM could not be acquired as the dissociation is too slow (*K*_dis_ < 10^–7^). Besides, no interaction of CdTe QDs and CdTe/ZnS QDs with uncoated streptavidin (SA) biosensor tips during the association period were confirmed and the streptavidin itself showed no cross reactivity with CdTe QDs and CdTe/ZnS QDs (data not shown). These results indicated that the assembling and aggregating of FIB, PLG and PTM with QDs led to the local concentrations increase and conformational changes in a concentration-dependent and time-dependent manner, that may be the trigger for the derangement of coagulation balance.

**Table 1:** Kinetic rate constants and fluorescence quenching rate constants for the interaction

Protein	CdTe QDs			CdTe/ZnS QDs		
	*K*_a_ (10^3^ /M s)	*K*_dis_ (10^−4^/s)	*K*_q_ (10^13^ L/M s)	*K*_a_ (10^3^ /M s)	*K*_dis_ (10^−4^/s)	*K*_q_ (10^13^ L/M s)
FIB	0.9420	4.414	0.8318	3.547	2.190	1.0960
PLG	1.703	< 0.001	0.9971	12.33	< 0.001	1.4560
PTM	1.440	< 0.001	1.6190	6.274	< 0.001	4.3970

**Fig. 5:**
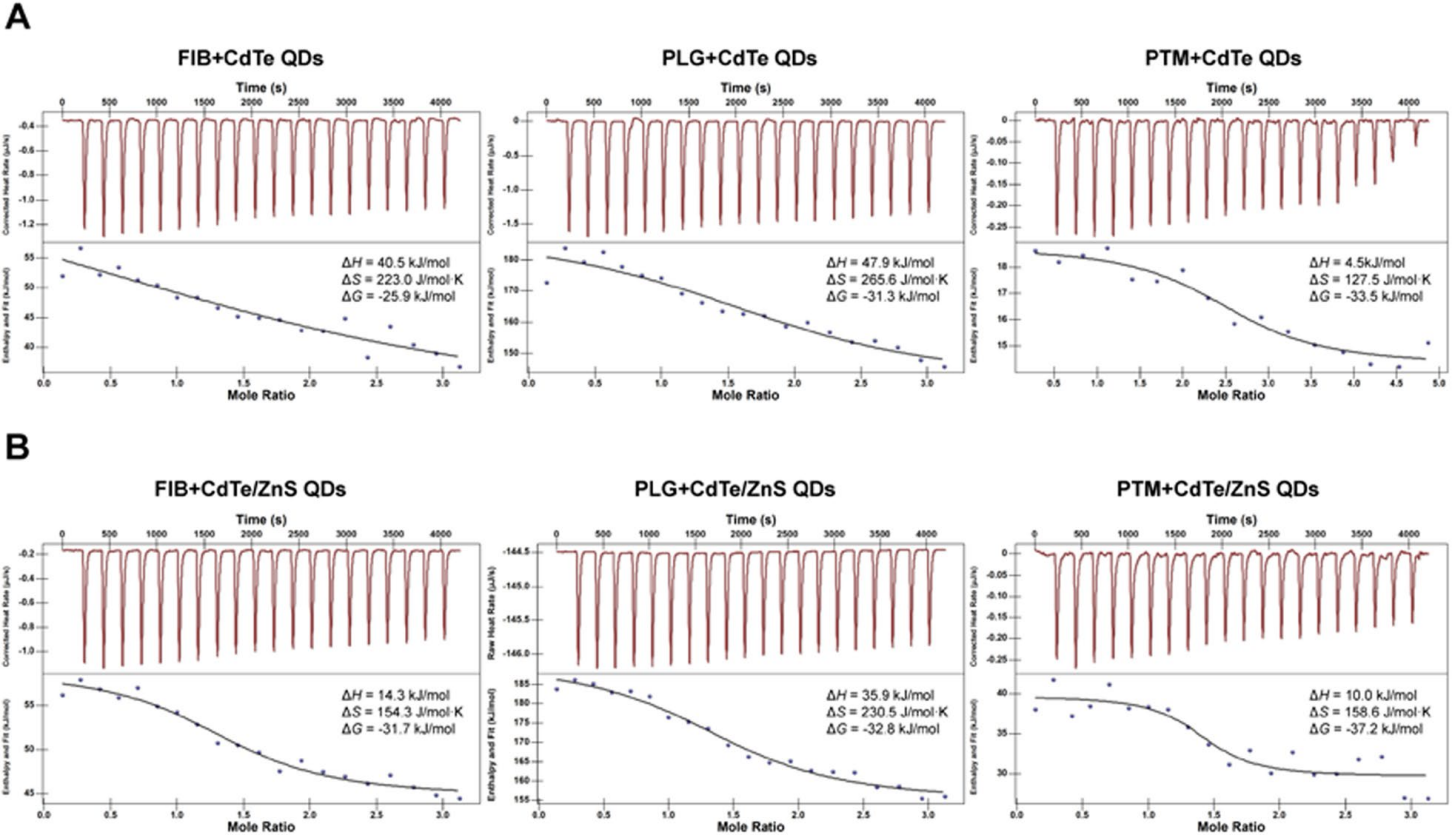
ITC curves and thermodynamic parameters of binding processes for FIB, PLG and PTM with CdTe QDs (**A**) and CdTe/ZnS QDs (**B**). The top panel presented the raw data collected at each injection, and the bottom panel presented the fitting curve of a plot of the heat flow per injection of QDs versus the molar ratio of protein. The concentrations of CdTe QDs and CdTe/ZnS QDs were 100 μmol/L, and the concentrations of FIB, PLG and PTM were 10 μmol/L

As a thermodynamic technique for directly measuring the heat released or absorbed during a biomolecular binding process, ITC assays could not only directly acquire the information of numbers of binding sites (*n*) and affinity constant (*K*_D_), but also simultaneously determine the enthalpy change (Δ*H*), gibbs free energy change (Δ*G*) and entropy change (Δ*S*) during the molecular titrating reaction process [32]. Figure 5 showed the raw data collected at each injection (top panel) and the fitting curve of a plot of the heat flow per injection of QDs versus the molar ratio of protein (bottom panel). As the thermodynamic parameters of the titration process showed in the Fig. 5, the binding processes between QDs and three proteins were endothermic and spontaneous because of an enthalpy gain (Δ*H* > 0) and negative Gibbs free energy change (Δ*G* < 0). For large proteins or protein conjugates which may behave as colloids, entropy-driven adsorption may be common [33]. In the presence of entropy-enthalpy compensation, an unfavorable enthalpy contribution (Δ*H* > 0) was partially offset by a large favorable entropy change (Δ*S* > 0), affording negative free energy (Δ*G* < 0) [34].

The interaction of biomacromolecules with ligands is a complex process that can be accompanied by various weak noncovalent interaction, including hydrophobic interaction, van der waals forces, multiple hydrogen bonds and electrostatic forces [35, 36]. The positive Δ*H* and Δ*S* suggested that the interaction between the QDs and three proteins were mainly driven by a hydrophobic interaction [37]. Hence, it was necessary to correlate the binding affinity with the hydrophobicity of protein molecules. The amino acid compositions were taken from the protein database and the hydrophobic index (*H*_index_) were calculated (Table 2) for all the proteins under consideration as detailed in reference [38]. These results yield that *K*_D_ for interaction of proteins and QDs followed the order of PTM > PLG > FIB, indicating the more positive is the *H*_index_, the higher is the protein-QDs binding. Besides, the numbers of binding sites for the interaction of proteins and CdTe QDs followed the order of FIB > PTM > PLG, while for the interaction of proteins and CdTe/ZnS QDs were similar. It was speculated that the smaller surface of CdTe QDs may reorganize upon the binding of the first protein molecule to expose more sites for interaction with the subsequent protein molecule. In comparison, the larger surface area of CdTe /ZnS QDs for binding may lead to only local reorganization of the surface groups and, thus, less impact on the subsequent binding [26].

**Table 2:** Binding affinity constants and numbers of binding sites for the interaction

Protein (*H*_index_)	CdTe QDs		CdTe/ZnS QDs	
	*K*_D_ (10^−6^ M)	*n*	*K*_D_ (10^−6^ M)	*n*
FIB (− 0.746)	28.74	2.643	2.809	1.411
PLG (− 0.688)	3.290	1.977	1.816	1.495
PTM (− 0.539)	1.336	2.511	0.2982	1.472

In the thermodynamic and kinetic processes of the interaction between proteins and QDs, *K*_a_ and *K*_D_ were larger for interaction of proteins with CdTe/ZnS QDs than CdTe QDs, that also explained why CE-ICP-MS found CdTe/ZnS QDs-protein conjugates formed faster than CdTe QDs-protein conjugates. These results were also in agreement with Bohidar et al. in that a threefold increment in *K*_D_ for BSA, HSA and *β*-Lg interacting with ZnSe@ZnS core–shell structure as compared to that of ZnSe core only QDs [39]. We speculated that it may be due to a coordinated binding with proteins by the Zn atoms at the surface of ZnS shell imperfections [40, 41]. Besides, the larger contact area between proteins and core–shell QDs compared with core only QDs may also strengthen the protein-nanoparticle interaction [42]. The higher affinity of CdTe/ZnS QDs to these coagulation-related proteins may increase their local concentrations in the blood, and activated more easily to induce significant coagulation cascade [24]. Moreover, the stronger binding with these coagulation-related proteins may also lead to a longer-term existance of CdTe/ZnS QDs in the blood than CdTe QDs. These may be the rational explanation for more stronger and last longer effect on coagulation factors and fibrinolytic factors after exposure of CdTe/ZnS QDs than CdTe QDs (Fig. 1).

### Fluorescence quenching of proteins and QDs

**Fig. 6:**
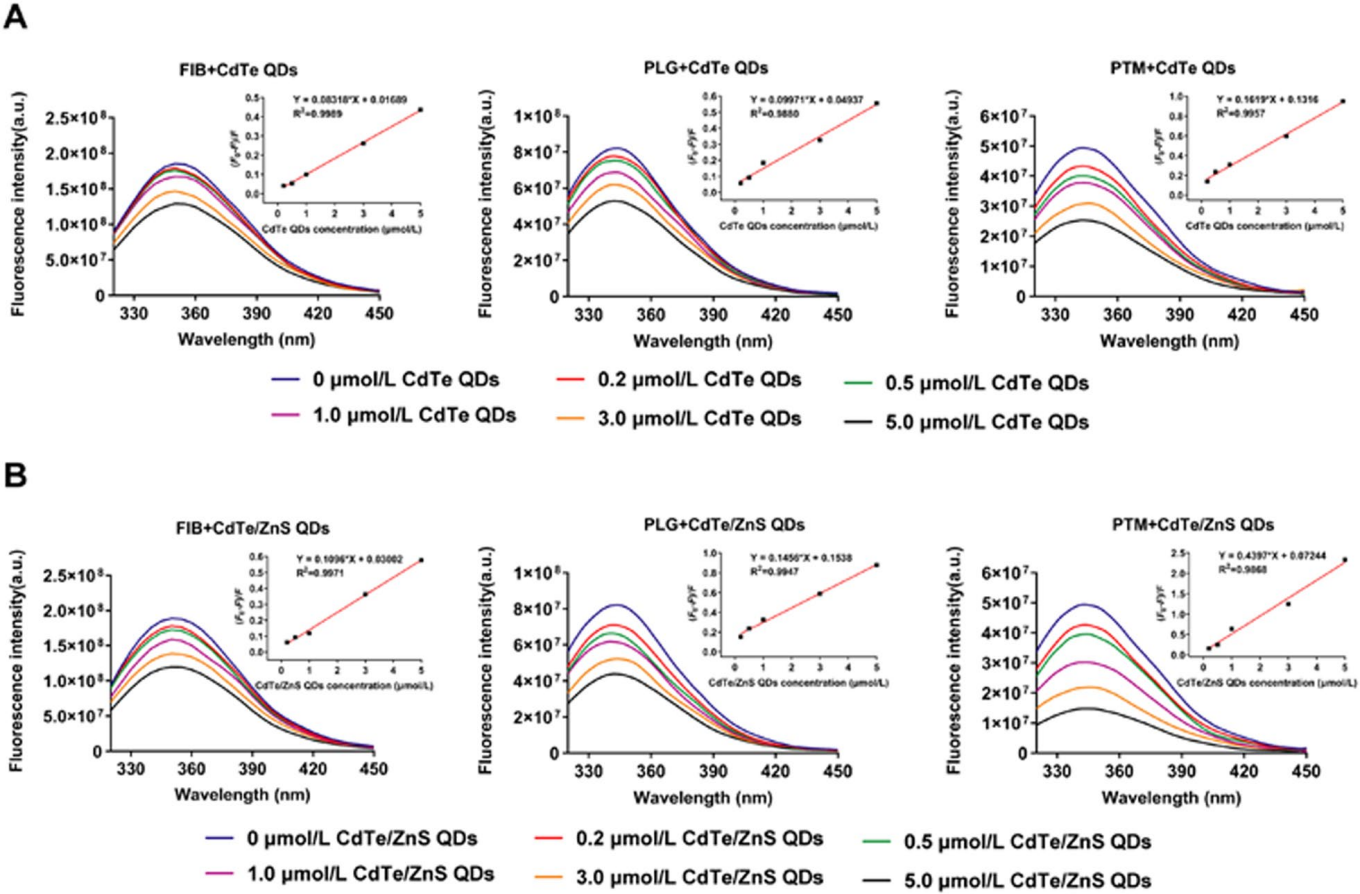
Fluorescence emission spectra of FIB, PLG and PTM in the presence of CdTe QDs (**A**) and CdTe/ZnS QDs (**B**). Inset: Stern–Volmer plot of (*F*_0_ − *F*)/*F* of FIB, PLG and PTM versus the different concentrations of QDs. The concentrations of FIB, PLG and PTM were 5.88 μmol/L, 2 μmol/L and 2 μmol/L, respectively

For understanding the binding mechanism of FIB, PLG and PTM with QDs, the fluorescence spectra of the proteins in the presence of different concentrations of CdTe QDs and CdTe/ZnS QDs were recorded at 280 nm excitation. From Fig. 6, it can be observed that the fluorescence quenching of three kinds of proteins occurred remarkably upon addition of CdTe QDs and CdTe/ZnS QDs. The fluorescence quenching of molecules can proceed via two major mechanisms, usually classified as static quenching and dynamic quenching. Dynamic quenching is due to the collision between the fluorophore and the quencher, whereas static quenching refers to the formation of the ground-state conjugates between them [43]. In order to obtain a clear insight into the quenching mechanism, the fluorescence quenching data were analyzed using the Stern–Volmer equation [44]

$$F_{0} /F = 1 + K_{SV} \left[ {{\text{QDs}}} \right] = \, 1 + K_{{\text{q}}} \tau_{0} \left[ {{\text{QDs}}} \right]$$

where *F*_0_ is the total fluorescence intensity of protein (in the absence of QDs), and *F* is the fluorescence intensity of protein at a specific QDs concentration. [QDs] represents the molar concentration of QDs, and *K*_sv_ refers to the Stern − Volmer quenching constant, a measure of the quenching efficiency. *K*_q_ accounts for the quenching rate constant, and *τ*_0_ (~ 10^–8^ s) is the average lifetime of protein in the absence of the quencher [45]. The inset in Fig. 6 demonstrated the linear dependence of (*F*_0_−*F*)/*F* of FIB, PLG and PTM as function of QDs concentration. As mentioned in Table 1, the obtained values of *K*_q_ are greater in comparison to the maximum scatter collision quenching constant of various quenchers (2.0 × 10^10^ L/M·s) [46, 47]. Therefore, the nature of quenching was not arising from dynamic collision, but was due to the formation of ground-state conjugates between QDs and the proteins subsequently causing static quenching [48]. Meanwhile, the *K*_q_ of proteins for interaction with both CdTe QDs and CdTe/ZnS QDs followed the order of PTM > PLG > FIB, which is also identical to the order of *K*_D_. For same protein, the *K*_q_ of CdTe/ZnS QDs was larger than CdTe QDs.

For exploring the effect of binding with proteins on the activity of QDs, the fluorescence spectra of CdTe QDs and CdTe/ZnS QDs after interaction with different concentrations of proteins were recorded at 360 nm excitation. As shown in Additional file 1: Fig. S7, the fluorescence intensity of CdTe QDs and CdTe/ZnS QDs decreased with increasing concentrations of PTM, PLG and FIB, indicating the electronic coupling and exciton energy transfer induced by interaction of QDs with proteins [49]. Notably, the fluorescence spectra of CdTe/ZnS QDs showed a slight blue-shift with increasing concentrations of PLG and a clear red-shift with increasing concentrations of FIB and PTM, respectively. These results also consist with those in CE-ICP-MS that the degradation in the presence of PLG and aggregation in the presence of these three proteins for CdTe/ZnS QDs.

### QDs-induced proteins conformational changes

**Fig. 7:**
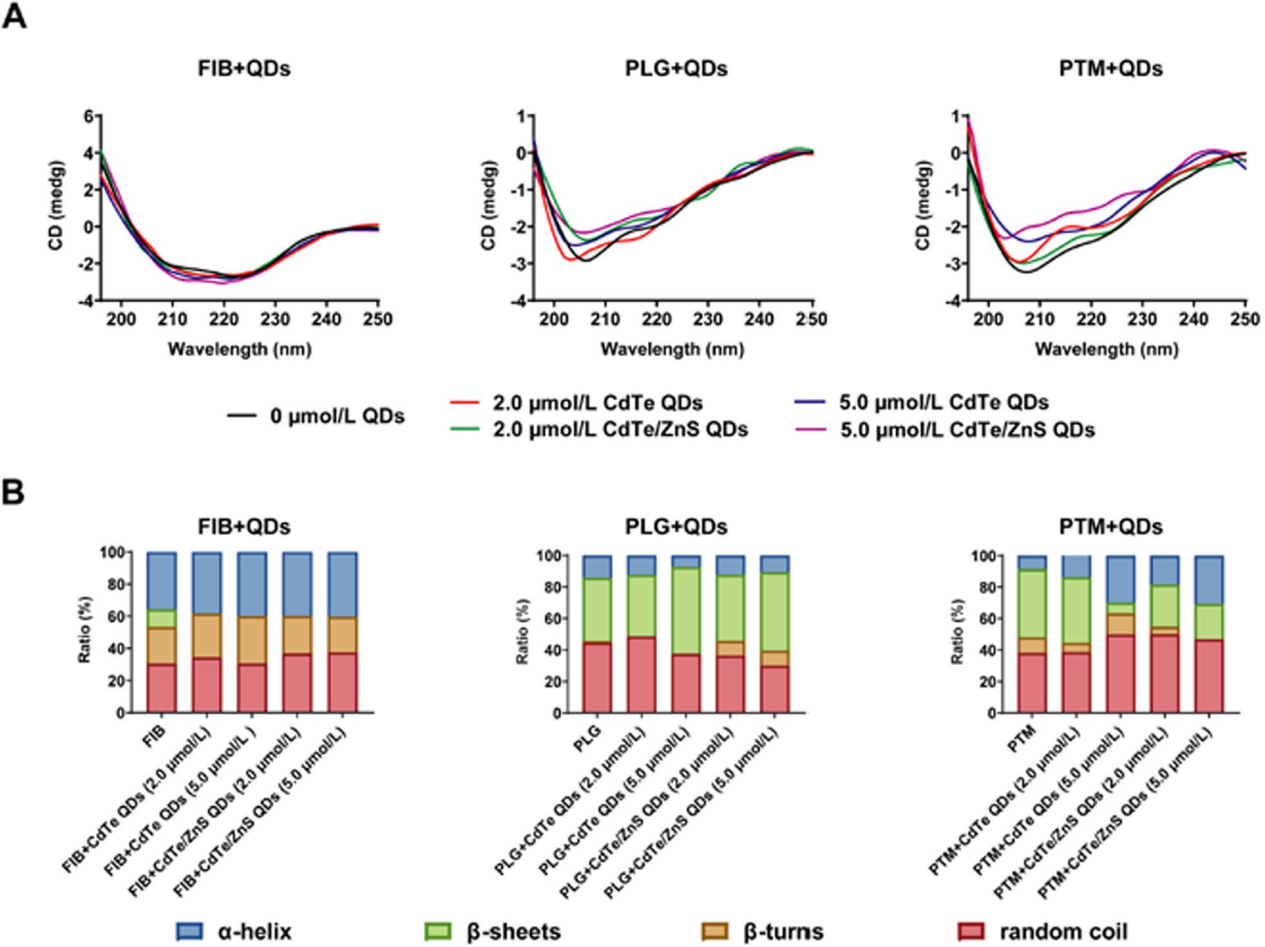
CD spectra for interaction of FIB, PLG and PTM with CdTe QDs and CdTe/ZnS QDs (**A**), and the ratios of α-helix, β-sheets, β-turns and random coil in the protein secondary structure in the presence of different concentrations of QDs (**B**). The concentrations of FIB, PLG and PTM were fixed at 3.0 μmol/L, 2.7 μmol/L and 3.7 μmol/L, respectively

In solution, proteins fluctuate between many different conformations due to structural flexibility and optimized interaction with the surface of metallic NPs by adapting their structures [50]. To examine the potential structural changes of proteins upon interaction with QDs, CD spectra experiments were performed. In the ultraviolet region, two negative bands characteristic of the typical α-helix structure of protein were observed in the CD spectra (208 and 222 nm). The negative peak at 208 nm is contributed from the π–π* transition, and 222 nm corresponds to the n → π* transition due to the peptide bond of α-helix [51]. As can be seen in Fig. 7, these proteins underwent conformational changes upon interaction with CdTe QDs and CdTe/ZnS QDs. For FIB, in the presence of CdTe QDs with increasing concentrations, the α-helix content increased more significantly but β-sheet content decreased less remarkably than with addition of CdTe/ZnS QDs. For PTM, the α-helix content increased after addition of both CdTe QDs and CdTe/ZnS QDs, the β-sheets decreased after addition of CdTe QDs and the β-turns decreased after addition of CdTe/ZnS QDs. For PLG, the α-helix content and random coils content decreased, and β-turns content increased with increasing concentration of both CdTe QDs and CdTe/ZnS QDs. β-sheets increased after the addition of CdTe QDs, but were not notably influenced after the addition of CdTe/ZnS QDs. For the secondary structure of proteins, the α-helix structure shows the order of protein molecules, while β-sheets, β-turns and random coils reflect the looseness of protein molecules. These results indicated that the refolding and conformational changes of FIB and PTM occurred in the presence of QDs. However, the hydrogen-bonding networks of PLG were destroyed, causing unfolding of PLG and the exposure of amino acid residues folded inside the protein to the solution. In addition, the different proportions of secondary structures of three proteins induced by CdTe QDs and CdTe/ZnS QDs were inconsistent and irregular. The conformational changes of proteins upon QDs-protein interaction may depend on both the physicochemical characteristics of QDs and the properties of specific protein, such as isoelectric point, molecular weight and hydrophobicity. It seemed that hard can be generally explained about how NPs adsorption induces the extent of protein conformational rearrangement, even under conditions where the protein binding constants are rather similar [21].

### Molecular docking analysis of L-GSH and L-Cys with proteins

**Fig. 8:**
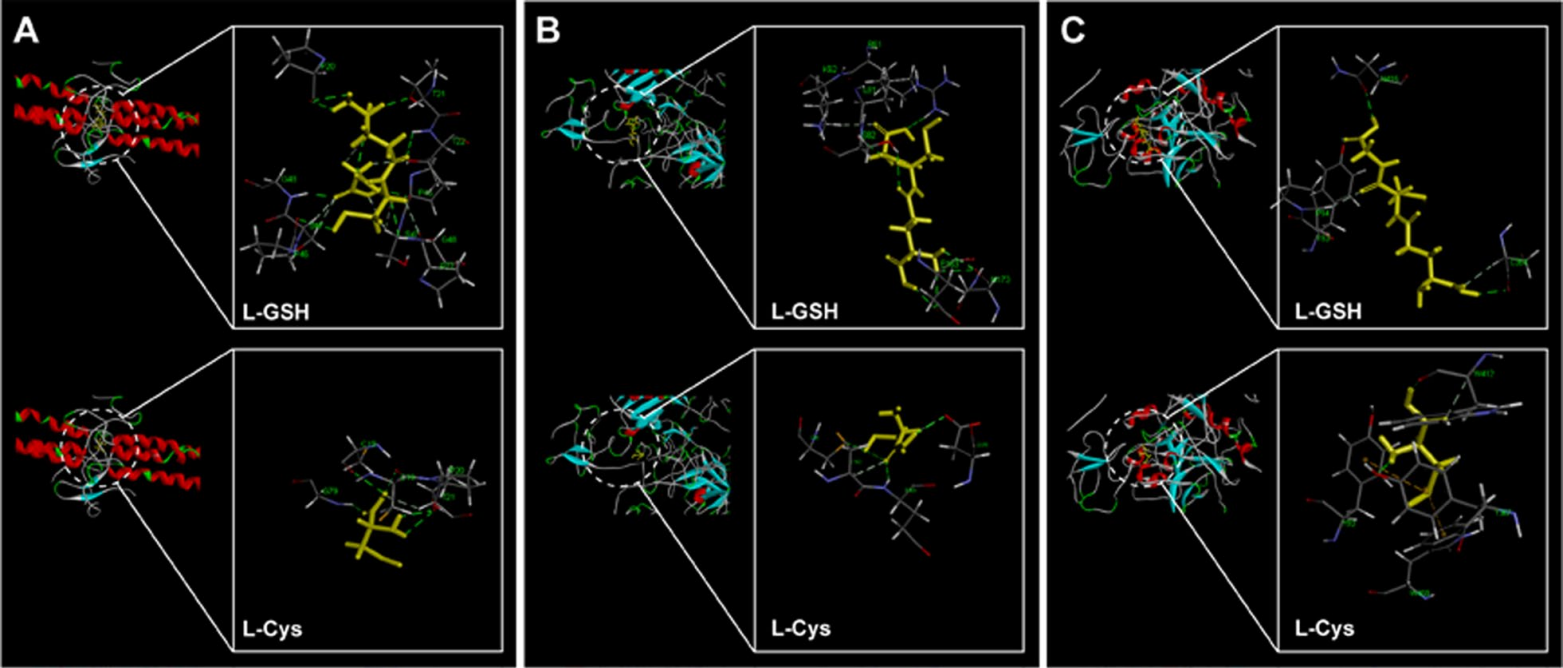
Molecular docking analysis of L-GSH and L-Cys with FIB (**A**), PLG (**B**) and PTM (**C**)

In order to further identify the specific binding sites and elucidate the binding forces between proteins and QDs, molecular docking analysis of the modified groups L-GSH and L-Cys of QDs to FIB, PLG and PTM receptors were performed. Among the 10 retrieved possible docking poses for each ligand, the sulfhydryl facing outward poses with highest scores were selected for further analysis, that can also be compatible when they linked with QDs. FIB is a soluble glycoprotein composed by disulfide-linked dimer of three nonidentical polypeptide chains, Aα, Bβ, and γ [52]. Previous results showed that multiple cavities more densely located in the N-terminal central nodule E-region of FIB corresponded to the thrombin binding-domain, which have critical importance for the blood coagulation process [53]. Therefore, the molecular docking simulations focused on the potential docking interaction between L-GSH and L-Cys with E-region of FIB. As shown in Fig. 8A, the binding occurred at polar uncharged threonine (Thr), glycine (Gly), Cys, serine (Ser) sites and nonpolar proline (Pro) site, and the binding formation involved hydrogen bonding and hydrophobic forces. Under the presence of ligands (L-GSH and L-Cys), some residues can be perturbed by modifications in the symmetry architecture of the Bβ-γ/Bβ-γ dimeric domain of E-region and could induce potential hematotoxicity effects-mediated like fibrinolysis [54].

PLG consists of seven domains: A N-terminal plasminogen-apple-nematode (PAN) domain, followed by five kringle domains (K1–K5) and the C-terminal catalytic trypsin-like serine protease (SP) domain [55]. Due to possessing lysine binding sites and the permanent exposure, K1 domain plays a key role in initial binding to lysine (Lys)-rich target surfaces like C-terminal fibrin monomers [56]. As shown in Fig. 8B, the ligands L-GSH and L-Cys were predicted to bind to the active pocket of K1 domain via hydrogen bonding and hydrophobic forces, and the binding sites included nonpolar leucine (Leu), negatively charged polar glutamic (Glu) and aspartic (Asp), positively charged polar argnine (Arg) and Lys, as well as polar uncharged Ser, asparagine (Asn) and Cys. The binding with active pocket of K1 domain may trigger the conformation transition of PLG into an open form, promoting the initial binding to Lys residues on target sites like fibrin [57].

PTM is a modular protein composed of the Gla domain, two kringle domains (K1 and K2), and the serine protease domain connected by three intervening linkers [58]. The intramolecular collapse of tryptophane93 (Tyr93) in kringle-1 onto Trp547 in the protease domain that obliterates access to the active site and protects the zymogen from autoproteolytic conversion to thrombin [59]. As shown in Fig. 8C, the ligands L-GSH and L-Cys were predicted to bind to nonpolar Pro, Trp, as well as polar uncharged tyrosine (Tyr), Asn, Cys via hydrogen bonding, hydrophobic forces and pi-sulfur bonding. It is worth noting that the interaction of L-GSH and L-Cys with Tyr93 may perturb the closed-open conformational equilibrium of PTM and promote the conversion of prothrombin to thrombin [59].

## Conclusions

Concerns about the hemocompatibility of QDs arising from possible introduction into the bloodstream by biomedical application and environmental exposure motivated this study. Obtained results evidenced that exposure to both CdTe QDs and CdTe/ZnS QDs could induce derangement of coagulation balance in rats in a dose-dependent manner and time-dependent manner. An in-depth evaluation on the interaction of QDs with key coagulation-related proteins indicated that the interactions of QDs with FIB, PLG and PTM can form stable QDs-protein conjugates. The affinity of proteins with QDs followed the order of PTM > PLG > FIB, and a higher for affinity and association rate with CdTe/ZnS QDs than CdTe QDs. Upon binding to QDs, the conformation transformation and the activation of proteins may interfere hemostasis and fibrinolysis processes. Altogether, the findings herein not only revealed that the interactions of QDs with coagulation-related proteins may be key contributors for interference of coagulation balance, but also provided the important hints for tuning the characteristics of QDs in regulating the conformational and functional changes of target biological molecules, that might show a promise in highly secured QDs design.

## Methods

### Preparation and characterization of QDs

L-GSH/ L-Cys capped CdTe QDs and CdTe/ZnS QDs were synthesized and purified as reported method in our previous work focused on good chemical stability and high photoluminescence quantum yield, and the concentrations of CdTe QDs and CdTe/ZnS QDs were calculated based on the molar mass of the Cd [60]. Zeta potential were determined by dynamic light scattering (DLS) (Malvern Nano-ZS90, UK), and sizes were measured by TEM (Jeol2100, Japan). Fluorescence emission spectra (F-4600, Hitachi, Japan) and UV–visible absorption spectra (UV-2600, Shimadzu, Japan) were recorded at 400–800 nm.

### Animals and exposure to QDs

Specific pathogen free male Wistar rats (8 weeks old) were purchased from Vital River Laboratory Animal Center (Beijing, China) and raised two per cage in the stable environmental conditions (24 ± 2 °C, 50–70% relative humidity, with 12 h light/12 h dark everyday) for at least seven days before experiments and water and food were provided ad libitum. All operations were carried out under the guidance of ARRIVE guidelines and EU Directive 2010/63/EU. All experimental procedures were approved by the Animal Experiments and Experimental Animal Welfare Committee of Capital Medical University (ethical review number: 2018-0003). Rats were randomly divided into seven groups with six rats in each group. CdTe QDs and CdTe/ZnS QDs were intravenously exposed to the six experimental groups with concentrations of 1.5 μmol/kg bw, 5 μmol/kg bw and 15 μmol/kg bw respectively, while the control group was given 0.9% saline [61]. At days 1, 3, 7 and 14 post-exposure, the rats were anesthetized with 3% pentobarbital sodium and blood samples were collected into tubes containing sodium citrate (Becton and Dickinson Company, UK).

### Coagulation assays

PT, APTT and TT were examined by SF-8100 fully automated coagulation analyzer (Succeder, China) within 2 h after plasma collection. FIB, thrombin–antithrombin complex (TAT), tissue factor (TF), active coagulation factor X (FXa), tissue factor pathway inhibitor (TFPI), anti-thrombin III (AT-III), PLG, t-PA (tissue plasminogen activator) and D-dimer were tested by ELISA kits (Cusabio, China).

### CE-ICP-MS analyses

CE-ICP-MS analyses were performed on a CE 7100 system (Agilent Technologies, USA) coupled to a 8800 ICP-MS spectrometer with an octopole reaction system working in He mode (Agilent Technologies, USA) through a microconcentric CE ESI SprayerIInebulizer (Agilent Technologies, USA). 1 μg/mL indium standard solution (Agilent Technologies, USA) was used as a make-up solution to control the stability of hyphenation performance and the efficiency of nebulization. The mass isotopes of ^111^ Cd, ^125^Te, and ^66^ Zn were monitored in order to observe the binding of QDs with proteins.

Prior to first use, a new fused-silica capillaries (i.d. 75 μm; length 70 cm) were initially by flushing with 1 mol/L NaOH for 10 min, maintaining for 10 min, ultrapure water for 10 min, and the running buffer for 20 min. Between each sample run, the capillaries were re-conditioned by rinsing with 0.1 mol/L NaOH for 3 min, ultrapure water for 3 min and the running buffer for 3 min. All the rinses were performed using a pressure drop of 935 mbar. Samples consisted of 0.1% dimethyl sulfoxide which served as a marker of electroosmosis. All solutions to be introduced into the capillary were filtered through a 0.22 μm syringe filter.

The experiments were performed with a separation voltage of 15 kV and sample loading at 35 mbar for 5 s. The ICP-MS was operated under Helium (He) gas mode, the details of optimized operation conditions are listed in Table 3. In order to maintain the stability of QDs and secure the conjugates formed under approximate physiological conditions from pH-induced changes in the CE system, phosphate buffer (10 mM, pH 8.0) was chosen as sample buffer solution. Meanwhile, the borate buffer solution (20 mM, pH 9.0) was found to be the most suitable electrophoretic buffers as affording the highest signals of analytes under scrutiny as well as superior separation conditions with respect to peak shape and migration time. The concentrations of FIB, PLG and PTM were selected based on their concentrations in plasma [13, 14, 54]. The protein concentrations in the following sections of experimental methods were also selected based on this. Since the lower concentration of QDs than that of proteins caused unstable electroosmotic flow, the concentration of QDs referred to our previous work for stable signal intensity and reproducibility of migration time [60]. The interaction time of QDs and proteins referred to the studies for formation of protein corona of QDs in the literatures [62, 63].

**Table 3:** Operation conditions of ICP-MS

Parameters	Value
Signal collection mode	TRA
RF power	1550 W
Make up gas (Ar) flow rate	1.35 L/min
Collision cell gas (He) flow rate	4.0 L/min
Peristaltic pump speed	0.1 rps
Nebulization room temperature	2 °C
Sampling depth (mm)	8.0 mm
Energy discrimination	− 4.0 V

### BLI and ITC assays

BLI experiments were performed in a blank 96 well plates (NuncF96 MicroWell™ Plates, Thermo Fisher Scientific, Germany) by Octet RED96e System (Sartorius FortéBio, CA). Prior to each assay, streptavidin (SA) biosensor tips (Sartorius FortéBio, CA) were pre-wetted in 200 μL PBS for at least 10 min. First, biotinylation of FIB, PLG and PTM were performed according to the manufacturer’s protocol (Genemore, China). Subsequently, kinetic assays steps were performed as follows: (1) begin with a brief baseline (60 s) in PBS to acquire initial signals; (2) immobilize the biotinylated proteins (biotin-FIB or biotin-PLG or biotin-PTM) on the sensors to obtain a loading signal increased by approximately 1 nm.; (3) establish a second baseline in PBS (120 s) to wash away non-immobilized biotinylated proteins from the sensors and establish new baseline signals; (4) associate the immobilized proteins with CdTe QDs or CdTe/ZnS QDs for 180 s at the concentrations of 0.1875 μmol/L, 0.375 μmol/L, 0.75 μmol/L, 1.5 μmol/L and 3 μmol/L; (5) dissociation of binding QDs and proteins in PBS for 180 s. The association and dissociation responses were baseline corrected and globally fitted to a simple 1:1 Langmuir model to calculate association rate constant (*K*_a_) and dissociation rate constant (*K*_d_) by Octet software (Sartorius FortéBio, CA).

ITC experiments were performed on Affinity ITC system (TA, USA). The titration experiment involved 20 injections (2 μL per injection) of QDs (QDs concentration = 100 μmol/L) at 200 s intervals was then titrated into the sample cell (volume = 350 μL) containing the buffer-matched protein solution (protein concentration = 10 μmol/L). The reference cell was filled with ultrapure water. During the experiment, the sample cell was stirred continuously at 125 rpm. The heat profile of protein and QDs dilution in the buffer alone was subtracted from the titration data (both normalized to zero). The data were analyzed to determine the numbers of binding sites (*n*), affinity constant (*K*_d_), and Δ*H*, Δ*S*, Δ*G* et al. thermodynamic parameters of the reaction on NanoAnalyze software (TA, USA).

### Fluorescence quenching measurements

Fluorescence quenching of proteins induced by QDs were recorded on SpectraMax i3x (Molecular Devices, USA) in the wavelength range of 290–450 nm upon excitation wavelength at 280 nm. The concentrations of FIB, PLG and PTM were 5.88 μmol/L, 2 μmol/L and 2 μmol/L, respectively. The concentrations of QDs interacting with these three proteins were fixed as 0.2 μmol/L, 0.5 μmol/L, 1.0 μmol/L, 3.0 μmol/L and 5.0 μmol/L for consistency of comparison. At the highest concentration of QDs, the molar ratio of protein/QDs was about 1:1 to 1:2, closing to the binding ratio of protein/QDs in the results of ITC.

Fluorescence quenching of QDs after binding to proteins were recorded in the wavelength range of 480–700 nm upon excitation wavelength at 360 nm. The concentrations of proteins were fixed as 0.2 μmol/L, 0.5 μmol/L, 1 μmol/L and 2 μmol/L. The concentrations of CdTe QDs and CdTe/ZnS QDs were also selected as 5.0 μmol/L based on the binding ratio of protein/QDs.

### CD spectra assays

CD spectra were recorded on Jasco J-810 spectropolarimeter with a scan rate of 200 nm/min. The wavelength was set from 190 to 250 nm with the interval of 0.2 nm using 1 mm quartz cells. The bandwidth was 1 nm. Each spectra represented an average scan result three times and was baseline corrected by subtracting the blank spectra of the corresponding phosphate buffer solution (10 mM, pH 8.0). The experiments were performed at 25 °C. The test solutions for CD were prepared by mixing the protein and QDs in phosphate buffer solution (10 mM, pH 8.0). In order to satisfy the UV–Vis absorbance of QDs-protein conjugates in the range of 0.6 to 1.2 to obtain the best ratio of signal to noise (S/N) for CD spectra, the concentrations of FIB, PLG and PTM were fixed at 3.0 μmol/L, 2.7 μmol/L and 3.7 μmol/L. The concentrations of QDs were fixed as 2.0 μmol/L and 5.0 μmol/L based on the binding molar ratio of protein/QDs about 1:1 to 1:2 in the results of ITC.

### Molecular docking simulation

In order to perform molecular docking simulation, the 3D crystal structures of FIB (PDB: 3GHG), PLG (PDB: 4DUR) and PTM (PDB: 6BJR) were obtained from the Protein Data Bank (www.rcsb.org). Water molecules and co-crystallized ligands were deleted and the protein structures were then prepared by the Prepare Protein protocol using the Discovery Studio (version3.5) software package. This was accomplished by insertion of missing atoms in incomplete residues, modelling of missing loop regions, deletion of alternate conformations (disorder), standardization of atom names, and protonation of titratable residues [64]. The ligands L-GSH and L-Cys were docked into the active pockets of FIB, PLG and PTM receptors to predict their binding sites and interaction forces.

### Statistical analysis

Statistical analysis was performed using two-way ANOVA with Bonferroni’s multiple comparison. The data were expressed as mean ± standard deviation (SD). Differences between groups with *P* values ≤ 0.05 were considered significant.

## Supplementary Information

** Additional file 1: A novel insight into mechanism of derangement of coagulation balance: interactions of quantum dots with coagulation-related proteins**. **Fig. S1.** UV-Vis absorption spectra and fluorescence emission spectra of CdTe QDs and CdTe/ZnS QDs. **Fig. S2.** TEM images of CdTe QDs and CdTe/ZnS QDs.**Fig. S3.** Effects of CdTe QDs and CdTe/ZnS QDs on coagulation function at three points-in-time. **Fig. S4.** Effects of CdTe QDs and CdTe/ZnS QDs on coagulation factors at three points-in-time. **Fig. S5.** Effects of CdTe QDs and CdTe/ZnS QDs on fibrinolytic factors at three points-in-time. **Fig. S6.** Effects of CdTe QDs and CdTe/ZnS QDs on anticoagulation factors at three points-in-time. **Fig. S7.** Fluorescence emission spectra of CdTe QDs and CdTe/ZnS QDs in the presence of FIB, PLG and PTM.

## Data Availability

The datasets used and/or analyzed during the current study are available from the corresponding author on reasonable request.

## Abbreviations

TAT: Thrombin–antithrombin complex
TF: Tissue factor
FXa: Active coagulation factor X
TFPI: Tissue factor pathway inhibitor
AT-III: Anti-thrombin III
t-PA: Tissue plasminogen activator

## References

[1] Hu L, Zhong H, He Z (2019). The cytotoxicities in prokaryote and eukaryote varied for CdSe and CdSe/ZnS quantum dots and differed from cadmium ions. Ecotox Environ Safe.

[2] Galeone A, Vecchio G, Malvindi MA, Brunetti V, Cingolani R, Pompa PP (2012). In vivo assessment of CdSe-ZnS quantum dots: coating dependent bioaccumulation and genotoxicity. Nanoscale.

[3] Sukanya D, Nathan DMGT, Mahesh R, Sagayaraj P (2019). Comparative studies on the aqueous synthesis and biocompatibility of L-cysteine and mercaptopropionic acid capped CdSe/CdS/ZnS core/shell/shell quantum dots. J Nanosci Nanotechnol.

[4] Liang X, Wu T, Wang Y, Wei T, Zou L, Bai C (2020). CdTe and CdTe@ZnS quantum dots induce IL-beta-mediated inflammation and pyroptosis in microglia. Toxicol Vitro.

[5] Lv C, Zhang T, Lin Y, Tang M, Zhai C, Xia H (2019). Transformation of viral light particles into near-infrared fluorescence quantum dot-labeled active tumor-targeting nanovectors for drug delivery. Nano Lett.

[6] Zhang Y, Malekjahani A, Udugama BN, Kadhiresan P, Chen H, Osborne M (2021). Surveilling and tracking COVID-19 patients using a portable quantum dot smartphone device. Nano Lett.

[7] Sanchez-Solis A, Esparza D, Orona-Navar A, Torres-Castro A, Manuel Rivas J, Ornelas-Soto N (2021). Light-emitting diodes based on quaternary CdZnSeS quantum dots. J Lumin.

[8] Ji X, Peng F, Zhong Y, Su Y, He Y (2014). Fluorescent quantum dots: Synthesis, biomedical optical imaging, and biosafety assessment. Colloid Surface B.

[9] Zhou F, Li Z, Chen H, Wang Q, Ding L, Jin Z (2020). Application of perovskite nanocrystals (NCs)/quantum dots (QDs) in solar cells. Nano Energy.

[10] Geys J, Nemmar A, Verbeken E, Smolders E, Ratoi M, Hoylaerts MF (2008). Acute toxicity and prothrombotic effects of quantum dots: impact of surface charge. Environ Health Persp.

[11] Samuel SP, Santos-Martinez MJ, Medina C, Jain N, Radomski MW, Prina-Mello A (2015). CdTe quantum dots induce activation of human platelets: Implications for nanoparticle hemocompatibility. Int J Nanomed.

[12] Maguire CM, Lavin M, Doyle M, Byrne M, Prina-Mello A, O'Donnell JS (2018). The anticoagulant properties of cadmium telluride quantum dots. J Interdiscip Nanomed.

[13] Ponting CP, Marshall JM, Cederholmwilliams SA (1992). Plasminogen—a structural review. Blood Coagul Fibrin.

[14] Lancellotti S, Basso M, De Cristofaro R (2013). Congenital prothrombin deficiency: an update. Semin Thromb Hemost.

[15] Miles LA, Parmer RJ (2013). Plasminogen receptors: the first quarter century. Semin Thromb Hemost.

[16] Iwaki T, Malinverno C, Smith D, Xu Z, Liang Z, Ploplis VA (2010). The generation and characterization of mice expressing a plasmin-inactivating active site mutation. J Thromb Haemost.

[17] Gonzalez-Durruthy M, Scanavachi G, Rial R, Liu Z, Cordeiro MNDS, Itri R (2019). Structural and energetic evolution of fibrinogen toward to the betablocker interactions. Int J Biol Macromol.

[18] Wang J, Zheng X, Zhang H (2019). Exploring the conformational changes in fibrinogen by forming protein corona with CdTe quantum dots and the related cytotoxicity. Spectrochim Acta Part A Mol Biomol Spectrosc.

[19] Wang Z, Wang C, Liu S, He W, Wang L, Gan J (2017). Specifically formed corona on silica nanoparticles enhances transforming growth factor β1 activity in triggering lung fibrosis. ACS Nano.

[20] Lozano Fernández T, Dobrovolskaia M, Camacho T, González Fernández Á, Simón VR (2019). Interference of metal oxide nanoparticles with coagulation cascade and interaction with blood components. Part Part Syst Char.

[21] Lacerda SHDP, Park JJ, Meuse C, Pristinski D, Becker ML, Karim A (2010). Interaction of gold nanoparticles with common human blood proteins. ACS Nano.

[22] Huang H, Lai W, Cui M, Liang L, Lin Y, Fang Q (2016). An evaluation of blood compatibility of silver nanoparticles. Sci Rep UK.

[23] Yu WW, Qu LH, Guo WZ, Peng XG (2003). Experimental determination of the extinction coefficient of CdTe, CdSe, and CdS nanocrystals. Chem Mater.

[24] Simak J, De Paoli S (2017). The effects of nanomaterials on blood coagulation in hemostasis and thrombosis. Wiley Interdiscip Rev Nanomed Nanobiotechnol.

[25] Wang J, Li J, Teng Y, Hu W, Chai H, Li J (2014). Studies on multivalent interactions of quantum dots-protein self-assemble using fluorescence coupled capillary electrophoresis. J Nanopart Res.

[26] Li N, Zeng S, He L, Zhong W (2010). Probing nanoparticle−protein interaction by capillary electrophoresis. Anal Chem.

[27] Legat J, Matczuk M, Timerbaev A, Jarosz M (2017). CE Separation and ICP-MS detection of gold nanoparticles and their protein conjugates. Chromatographia.

[28] Matczuk M, Legat J, Timerbaev AR, Jarosz M (2016). A sensitive and versatile method for characterization of protein-mediated transformations of quantum dots. Analyst (London).

[29] Qu Y, Li W, Zhou Y, Liu X, Zhang L, Wang L (2011). Full assessment of fate and physiological behavior of quantum dots utilizing caenorhabditis elegans as a model organism. Nano Lett.

[30] Wang TX, Sridhar R, Korotcov A, Ting AH, Francis K, Mitchell J (2011). Synthesis of amphiphilic triblock copolymers as multidentate ligands for biocompatible coating of quantum dots. Colloid Surface A.

[31] Jayaram DT, Pustulka SM, Mannino RG, Lam WA, Payne CK (2018). Protein corona in response to flow: effect on protein concentration and structure. Biophys J.

[32] Zhang X, Zhang J, Zhang F, Yu S (2017). Probing the binding affinity of plasma proteins adsorbed on Au nanoparticles. Nanoscale.

[33] Huang R, Carney RP, Stellacci F, Lau BLT (2013). Protein–nanoparticle interactions: the effects of surface compositional and structural heterogeneity are scale dependent. Nanoscale.

[34] Eren NM, Narsimhan G, Campanella OH (2016). Protein adsorption induced bridging flocculation: the dominant entropic pathway for nano-bio complexation. Nanoscale.

[35] Paul BK, Ray D, Guchhait N (2013). Unraveling the binding interaction and kinetics of a prospective anti-HIV drug with a model transport protein: results and challenges. Phys Chem Chem Phys.

[36] Zhao X, Lu D, Liu QS, Li Y, Feng R, Hao F (2017). Hematological effects of gold nanorods on erythrocytes: hemolysis and hemoglobin conformational and functional changes. Adv Sci.

[37] Ross PD, Subramanian S (1981). Thermodynamics of protein association reactions: forces contributing to stability. Biochemistry.

[38] Rawat K, Bohidar HB (2012). Universal charge quenching and stability of proteins in 1-methyl-3-alkyl(hexyl/octyl) imidazolium chloride ionic liquid solutions. J Phys Chem B.

[39] Mir IA, Rawat K, Bohidar HB (2017). Interaction of plasma proteins with ZnSe and ZnSe@ZnS core-shell quantum dots. Colloids Surf A.

[40] Morgner F, Stufler S, Geißler D, Medintz IL, Algar WR, Susumu K (2011). Terbium to quantum dot FRET bioconjugates for clinical diagnostics: influence of human plasma on optical and assembly properties. Sensors.

[41] Simón-Vázquez R, Lozano-Fernández T, Peleteiro-Olmedo M, González-Fernández Á (2014). Conformational changes in human plasma proteins induced by metal oxide nanoparticles. Colloids Surf B.

[42] Yang B, Liu R, Hao X, Wu Y, Du J (2013). Effect of CdTe quantum dots size on the conformational changes of human serum albumin: results of spectroscopy and isothermal titration calorimetry. Biol Trace Elem Res.

[43] Ge B, Li Z, Yang L, Wang R, Chang J (2015). Characterization of the interaction of FTO protein with thioglycolic acid capped CdTe quantum dots and its analytical application. Spectrochim Acta A.

[44] Liang J, Cheng Y, Han H (2008). Study on the interaction between bovine serum albumin and CdTe quantum dots with spectroscopic techniques. J Mol Struct.

[45] Ding L, Zhou PJ, Li SQ, Shi GY, Zhong T, Wu M (2011). Spectroscopic studies on the thermodynamics of L-cysteine capped CdSe/CdS quantum dots-BSA interactions. J Fluoresc.

[46] Vaishanav SK, Korram J, Nagwanshi R, Ghosh KK, Satnami ML (2016). Adsorption kinetics and binding studies of protein quantum dots interaction: a spectroscopic approach. J Fluoresc.

[47] Naveenraj S, Raj MR, Anandan S (2012). Binding interaction between serum albumins and perylene-3,4,9,10-tetracarboxylate—a spectroscopic investigation. Dyes Pigments.

[48] Peng XG, Schlamp MC, Kadavanich AV, Alivisatos AP (1997). Epitaxial growth of highly luminescent CdSe/CdS core/shell nanocrystals with photostability and electronic accessibility. J Am Chem Soc.

[49] Koole R, Liljeroth P, Donega CDM, Vanmaekelbergh D, Meijerink A (2006). Electronic coupling and exciton energy transfer in CdTe quantum-dot molecules. J Am Chem Soc.

[50] Ozboyaci M, Kokh DB, Corni S, Wade RC (2016). Modeling and simulation of protein-surface interactions: achievements and challenges. Q Rev Biophys.

[51] Cooper TM, Woody RW (1990). The effect of conformation on the CD of interacting helices: a theoretical study of tropomyosin. Biopolymers.

[52] Liu Y, Tang X, Pei J, Zhang L, Liu F, Li K (2006). Gastrodin interaction with human fibrinogen: anticoagulant effects and binding studies. Chem Eur J.

[53] González-Durruthy M, Concu R, Vendrame LFO, Zanella I, Ruso JM, Cordeiro MNDS (2020). Targeting beta-blocker drug–drug interactions with fibrinogen blood plasma protein: a computational and experimental study. Molecules.

[54] González-Durruthy M, Rial R, Cordeiro MNDS, Liu Z, Ruso JM (2021). Exploring the conformational binding mechanism of fibrinogen induced by interactions with penicillin β-lactam antibiotic drugs. J Mol Liq.

[55] Rahi A, Dhiman A, Singh D, Lynn AM, Rehan M, Bhatnagar R (2018). Exploring the interaction between Mycobacterium tuberculosis enolase and human plasminogen using computational methods and experimental techniques. J Cell Biochem.

[56] Vali Z, Patthy L (1980). Essential carboxyl- and guanidino-group in the lysine-binding site of human plasminogen. Biochem Biophys Res Commun.

[57] Steinmetzer T, Pilgram O, Wenzel BM, Wiedemeyer SJA (2020). Fibrinolysis inhibitors: potential drugs for the treatment and prevention of bleeding. J Med Chem.

[58] Pozzi N, Bystranowska D, Zuo X, Di Cera E (2016). Structural architecture of prothrombin in solution revealed by single molecule spectroscopy. J Biol Chem.

[59] Chinnaraj M, Chen Z, Pelc LA, Grese Z, Bystranowska D, Di Cera E (2018). Structure of prothrombin in the closed form reveals new details on the mechanism of activation. Sci Rep.

[60] Meng P, Xiong Y, Wu Y, Hu Y, Wang H, Pang Y (2018). A novel strategy to evaluate the degradation of quantum dots: identification and quantification of CdTe quantum dots and corresponding ionic species by CZE-ICP-MS. Chem Commun.

[61] Hu Y, Xiong Y, Kong L, Huang P (2019). Effects of exposure to CdTe quantum dots on coagulation-related factors in mice. Asian J Ecotoxicol.

[62] Prapainop K, Wentworth P (2011). A shotgun proteomic study of the protein corona associated with cholesterol and atheronal-B surface-modified quantum dots. Eur J Pharm Biopharm.

[63] Pleskova SN, Bobyk SZ, Fomichev OI, Boryakov AV, Gorshakova EN (2020). Influence of quantum dots protein crown on the morphology and morphometric characteristics of lymphocytes. Bull Exp Biol Med.

[64] Wang S, Jiang J, Li R, Deng P (2020). Docking-based virtual screening of TβR1 inhibitors: evaluation of pose prediction and scoring functions. BMC Chem.

