# Supplementary material for: A novel insight into mechanism of derangement of coagulation balance: interactions of quantum dots with coagulation-related proteins
Source: Part Fibre Toxicol. 2022 Mar 8;19:17. doi: 10.1186/s12989-022-00458-x (PMC8903618; doi:10.1186/s12989-022-00458-x)
Supplement: Supplementary file 1 — Additional file 1: A novel insight into mechanism of derangement of coagulation balance: interactions of quantum dots with coagulation-related proteins. Fig. S1. UV-Vis absorption spectra and fluorescence emission spectra of CdTe QDs and CdTe/ZnS QDs. Fig. S2. TEM images of CdTe QDs and CdTe/ZnS QDs.Fig. S3. Effects of CdTe QDs and CdTe/ZnS QDs on coagulation function at three points-in-time. Fig. S4. Effects of CdTe QDs and CdTe/ZnS QDs on coagulation factors at three points-in-time. Fig. S5. Effects of CdTe QDs and CdTe/ZnS QDs on fibrinolytic factors at three points-in-time. Fig. S6. Effects of CdTe QDs and CdTe/ZnS QDs on anticoagulation factors at three points-in-time. Fig. S7. Fluorescence emission spectra of CdTe QDs and CdTe/ZnS QDs in the presence of FIB, PLG and PTM. [file 12989_2022_458_MOESM1_ESM.docx]

**Electronic Supporting Information**

**A novel insight into** **mechanism of** **derangement of coagulation balance: interactions of quantum dots with coagulation-related proteins**

Lingyan Zhang^1,2^, Yingting Wu^3^, Xingling Luo^1^, Tianjiang Jia^1^, Kexin Li^1^, Lihong Zhou^1^, Zhen Mao^1^ and Peili Huang^1,^^[[1]](#footnote-1)^

SI1. **Characterization of CdTe QDs and CdTe/ZnS QDs**


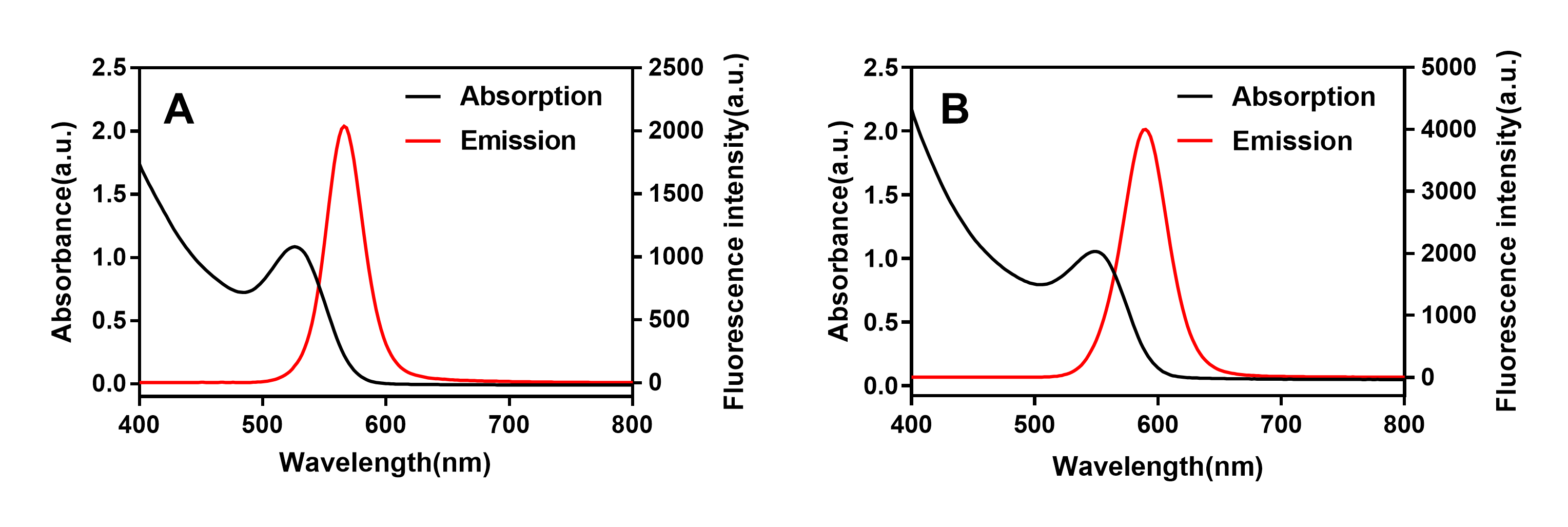


Fig. S1 UV-Vis absorption spectra and fluorescence emission spectra of (A) CdTe QDs and (B) CdTe/ZnS QDs.


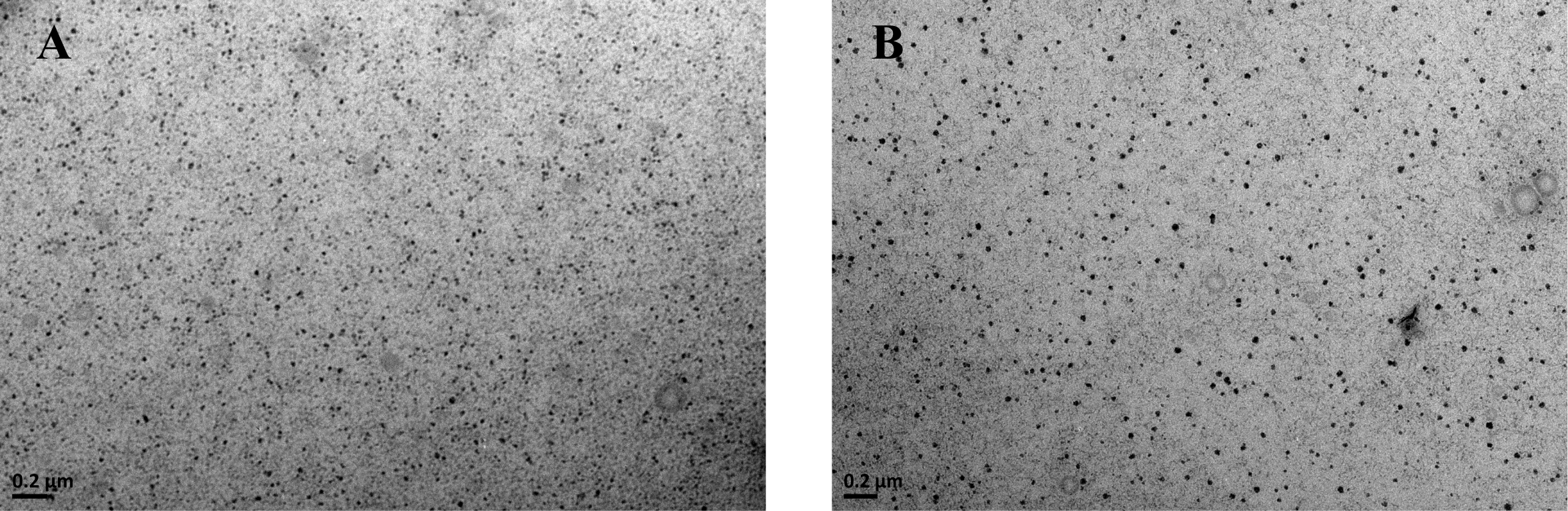


Fig. S2 TEM images of (A) CdTe QDs and (B) CdTe/ZnS QDs. Scale bar = 0.2 μm.

SI2. **QDs induced derangement of coagulation balance**

At day 3, day 7 and day 14, no statistically significant changes of PT, APTT and TT in rats were observed of both CdTe QDs-treated groups and CdTe/ZnS QDs-treated groups (Fig. S3).

For coagulation factors (Fig. S4), FXa still decreased in each dosage group of CdTe/ZnS QDs (*P*<0.05) at day 3. For fibrinolytic factors (Fig. S5), PLG decreased remarkably in 15 μmol/kg bw group of CdTe/ZnS QDs (*P*<0.001) at day 3, and the level of PLG is still lower in 5 μmol/kg bw group and 15 μmol/kg bw group of CdTe/ZnS QDs (*P*<0.01) at day 7. What’s more, the level of t-PA was still higher in 15 μmol/kg bw group of CdTe/ZnS QDs (*P*<0.001) at day3. For anticoagulation factors (Fig. S6), no significant changes were observed for tissue factor pathway inhibitor (TFPI) and antithrombin III (AT-III) in each dosage group of CdTe QDs and CdTe/ZnS QDs at each point in time of observation (*P*>0.05).


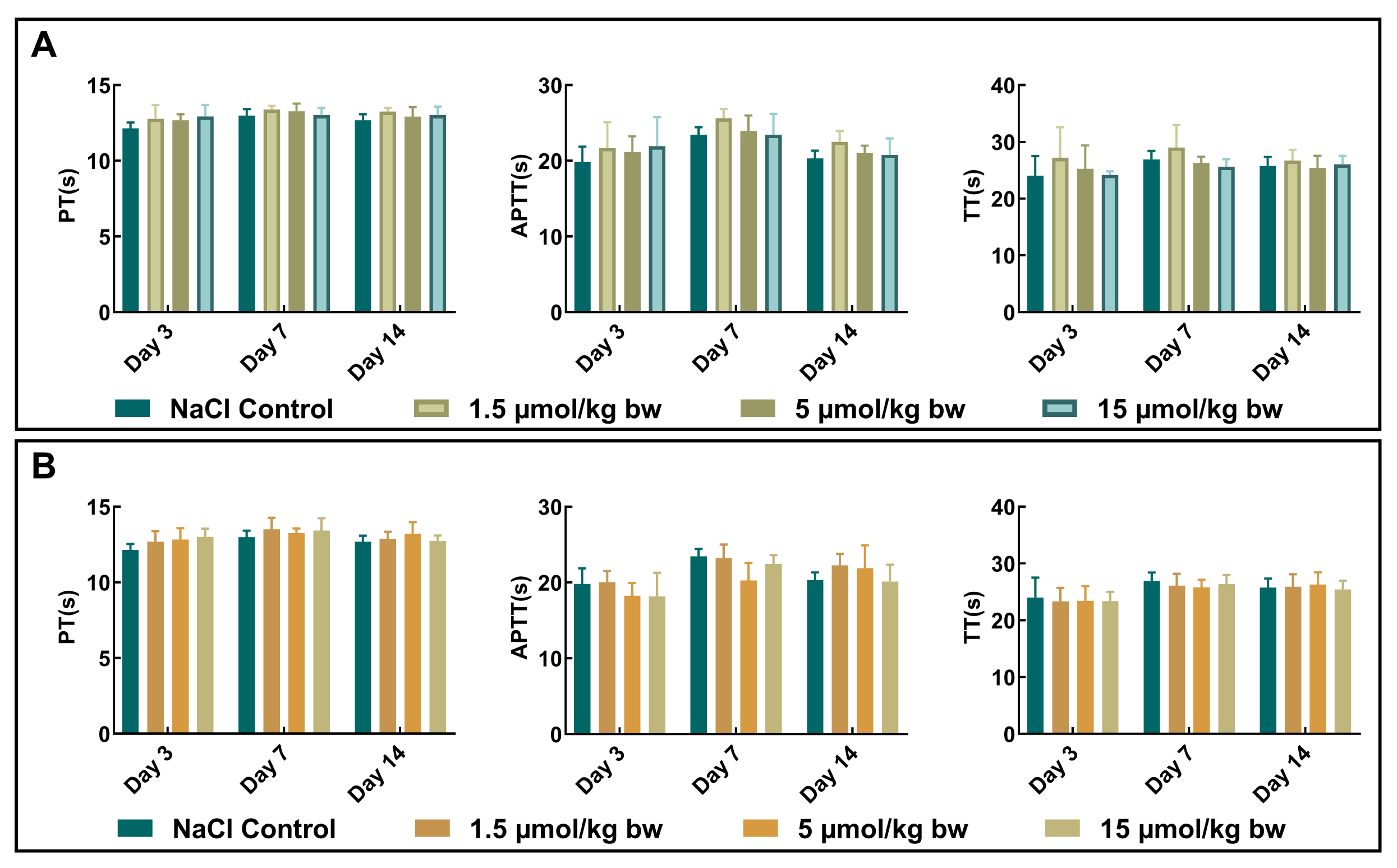


**Fig. S3** Effects of CdTe QDs (A) and CdTe/ZnS QDs (B) on coagulation function at three points-in-time. Each value represents the mean ± standard deviation (SD) (*n* = 6). Comparing to the values of control group and dose group by two-way ANOVA with Bonferroni’s multiple comparison test.

**
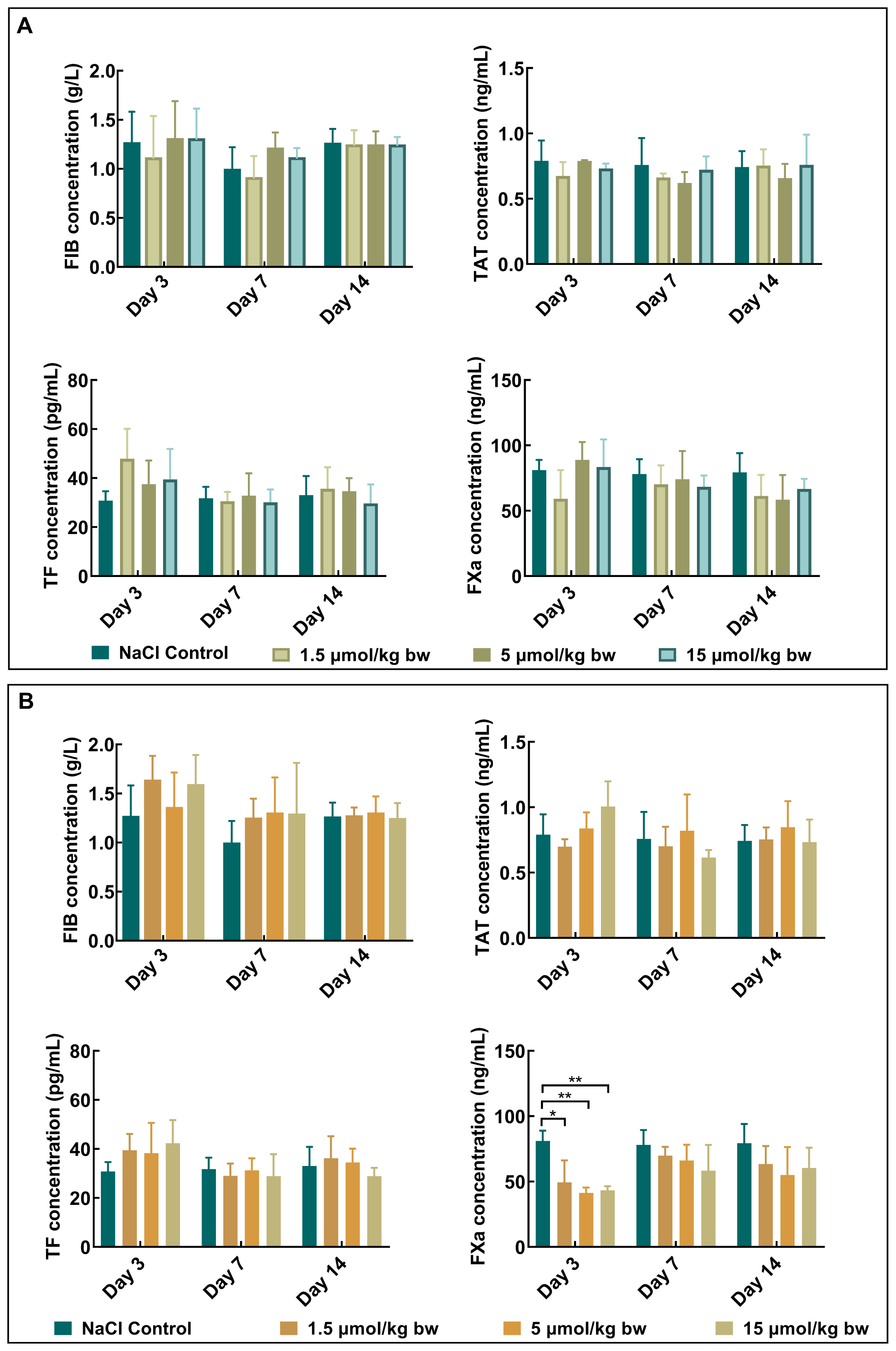
**

**Fig. S4** Effects of CdTe QDs (A) and CdTe/ZnS QDs (B) on coagulation factors at three points-in-time. Each value represents the mean ± standard deviation (SD) (*n* = 6). * *P* < 0.05, ** *P* < 0.01, comparing to the values of control group and dose group by two-way ANOVA with Bonferroni’s multiple comparison test.


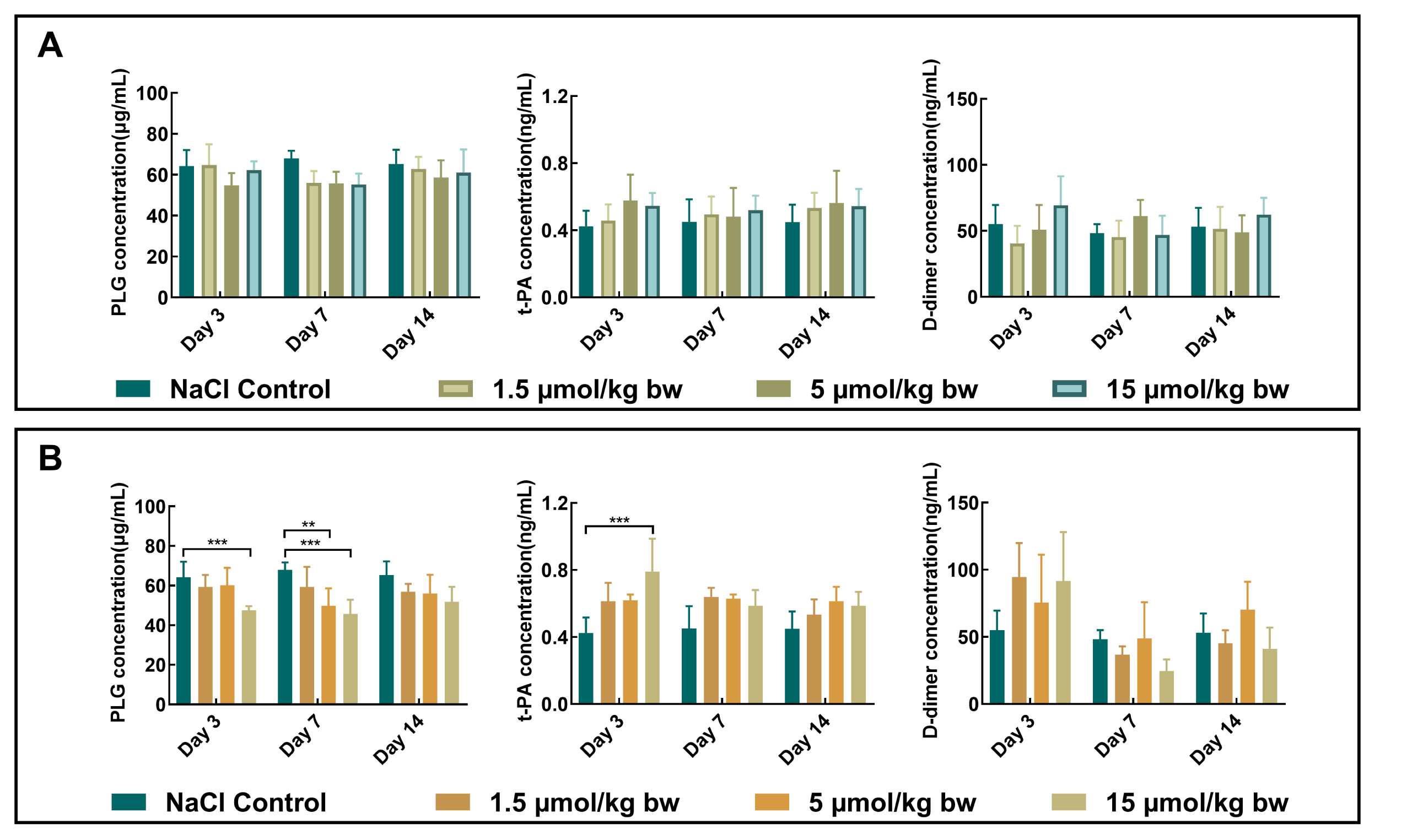


**Fig. S5** Effects of CdTe QDs (A) and CdTe/ZnS QDs (B) on fibrinolytic factors at three points-in-time. Each value represents the mean ± standard deviation (SD) (*n* = 6). ** *P* < 0.01, *** *P* < 0.001, comparing to the values of control group and dose group by two-way ANOVA with Bonferroni’s multiple comparison test.


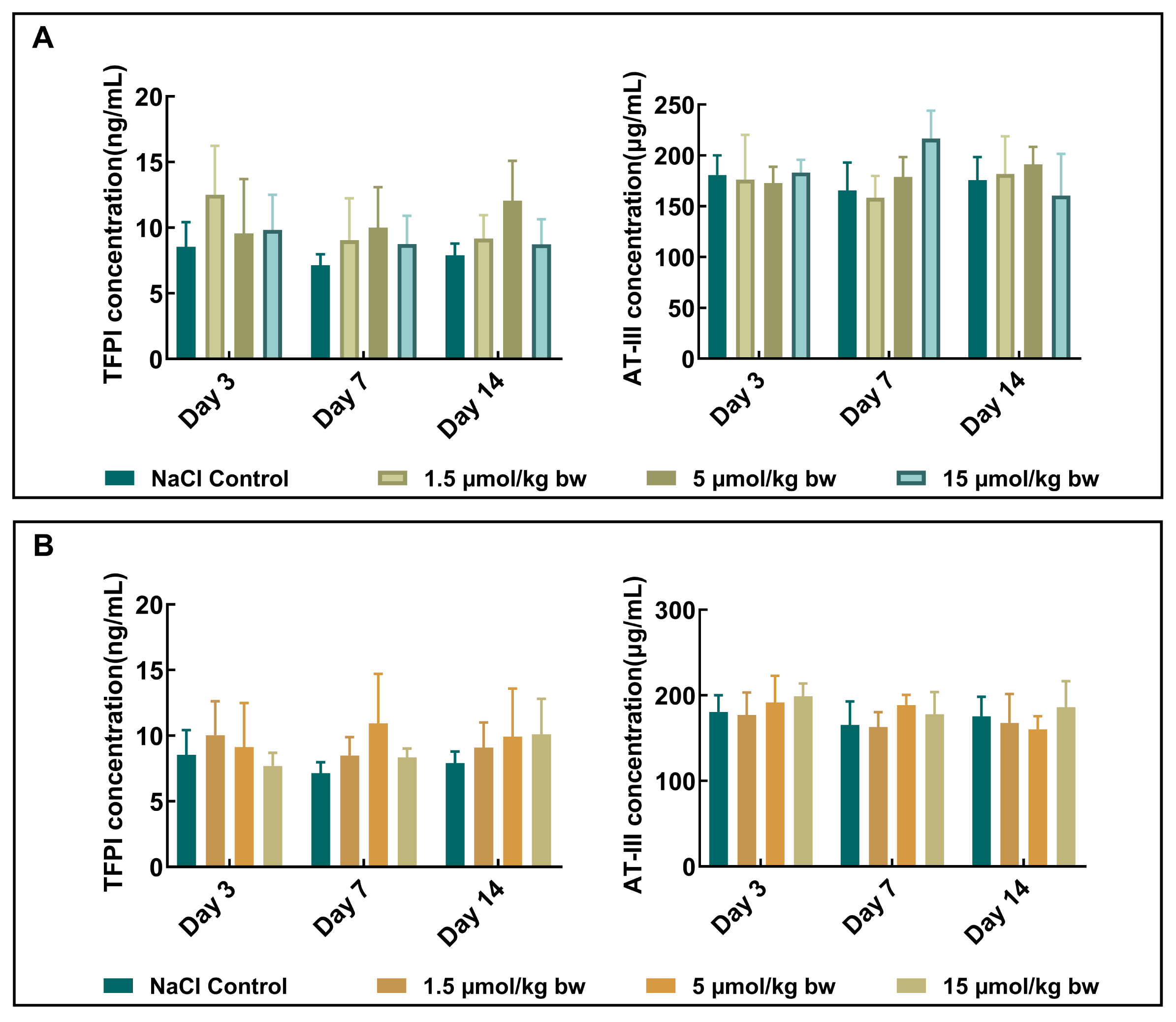


**Fig. S6** Effects of CdTe QDs (A) and CdTe/ZnS QDs (B) on anticoagulation factors at three points-in-time. Each value represents the mean ± standard deviation (SD) (*n* = 6). Comparing to the values of control group and dose group by two-way ANOVA with Bonferroni’s multiple comparison test.

**SI3. Fluorescence quenching of QDs**


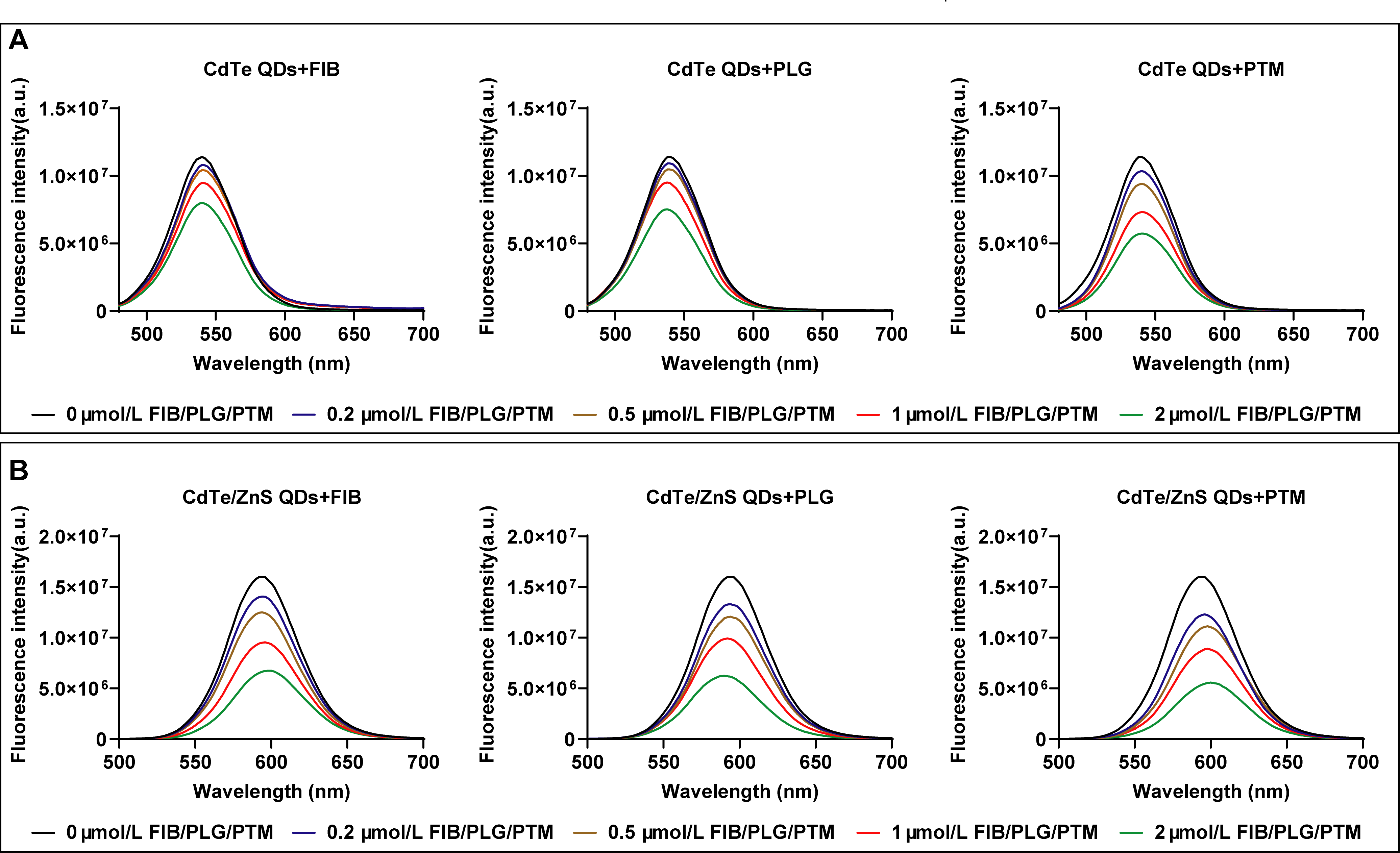


**Fig. S7** Fluorescence emission spectra of CdTe QDs (A) and CdTe/ZnS QDs (B) in the presence of FIB, PLG and PTM. The concentrations of FIB, PLG and PTM were 5.0 μmol/L.

1. Correspondence: [huangpl@ccmu.edu.cn](mailto:huangpl@ccmu.edu.cn)

   ^1^School of Public Health, Capital Medical University, No.10 Xitoutiao You An Men, Beijing 100069, China

   ^2^School of Public Health, Baotou Medical College, 31# Jianshe Road, Donghe District, Baotou 014040, China

   ^3^Core Facility Center, Capital Medical University, No.10 Xitoutiao You An Men, Beijing 100069, China [↑](#footnote-ref-1)
